# Breakdown of Chlorophyll in Higher Plants—Phyllobilins as Abundant, Yet Hardly Visible Signs of Ripening, Senescence, and Cell Death

**DOI:** 10.1002/anie.201508928

**Published:** 2016-02-26

**Authors:** Bernhard Kräutler

**Affiliations:** ^1^ Institute of Organic Chemistry & Center of Molecular Biosciences (CMBI)University of Innsbruck6020InnsbruckAustria

**Keywords:** biodegradation, chlorophyll, metabolism, natural product, plants

## Abstract

Fall colors have always been fascinating and are still a remarkably puzzling phenomenon associated with the breakdown of chlorophyll (Chl) in leaves. As discovered in recent years, nongreen bilin‐type Chl catabolites are generated, which are known as the phyllobilins. Collaborative chemical‐biological efforts have led to the elucidation of the key Chl‐breakdown processes in senescent leaves and in ripening fruit. Colorless and largely photoinactive phyllobilins are rapidly produced from Chl, apparently primarily as part of a detoxification program. However, fluorescent Chl catabolites accumulate in some senescent leaves and in peels of ripe bananas and induce a striking blue glow. The structural features, chemical properties, and abundance of the phyllobilins in the biosphere suggest biological roles, which still remain to be elucidated.

## 1 Introduction

The rejuvenating appearance of chlorophyll (Chl) in spring and the seemingly pompous disappearance of the green plant pigments in the autumnal foliage of deciduous trees and bushes are very colorful natural phenomena. The seasonal breakdown of Chl, in particular, has always been enchanting, and also most puzzling. Indeed, Chl metabolism is probably the most visual sign of life on Earth, even observable from outer space.^1^ It has been estimated that more than 1000 million tons of the green plant pigment are biosynthesized and degraded every year on Earth.^2^ Once, it was believed that colored products would result from Chl breakdown, similar to the bilins from the breakdown of heme,^3^ or to photo‐oxygenolysis products of Chl.^4^ Thus, all the early searches for Chl catabolites concentrated on the detection of such hypothetical colored leftovers of green Chl.^5^ As we now understand better, all of these endeavors were futile,1a, 6 and Chl seemed to disappear without leaving a trace.^2^ Colored Chl catabolites were, indeed, not detected in higher plants, until very recently.^7^

Only in the last 25 years^8^ has Chl breakdown in plants begun to reveal some of its molecular and cellular mysteries.1a, 9 An original breakthrough was achieved by the unambiguous identification and structure elucidation of colorless Chl catabolites from vascular plants,1a, 8, 10 thereby paving the way for fundamental insights into Chl breakdown.9a,9c, 11 In distantly related studies, Kishi, Shimomura, and co‐workers identified Chl‐derived tetrapyrroles as luminescent compounds from marine photoorganisms.^12^ However, as became apparent in the early 1990s, these linear tetrapyrroles^12^ differed basically from the Chl catabolites from higher plants.8a, 10

### 1.1 A Breakthrough: Identification of a First Colorless Chl Catabolite from Higher Plants

By comparison of the pigment patterns of natural, wild‐type senescent leaves of the grass *Festuca pratensis* and of barley (*Hordeum vulgare*) with the ones of corresponding “stay‐green” mutants (that do not degreen), Matile, Thomas et al. were able to identify several colorless compounds that accumulated in senescent wild‐type leaves, but were absent in the mutants.^5^, ^13^ Some of these colorless compounds were suspected to be products of Chl breakdown. Several of them decomposed readily into rust‐colored compounds and were called “rusty pigments”.^14^

“Rusty pigment 14” (**1**) was a major colorless fraction in extracts of senescent primary leaves of barley, and suspected to be a Chl catabolite.^14^, ^15^ ^14^C‐Labeled *δ*‐aminolevulinic acid, the biosynthetic precursor of the natural porphyrinoids, was incorporated into the “rusty pigment 14” fraction, thereby providing further support for its presumed role as a Chl catabolite.^15^ Indeed, **1** could be identified as a Chl‐derived linear tetrapyrrole8a by a combination of mass spectrometry^16^ as well as UV/Vis, CD, and NMR spectroscopy.^17^ It was characterized as an optically active metal‐free, colorless, and nonfluorescent linear tetrapyrrole with unconjugated pyrrole units. The linear tetrapyrrole **1** featured a Chl‐diagnostic cyclopentanone unit, annealed to the α‐ and β‐positions of a pyrrole ring, and carrying a methoxycarbonyl group. The colorless and nonfluorescent bilin‐type tetrapyrrole **1** was, thus, identified as the first nongreen Chl catabolite from higher plants.1a, 8a, 9a

**Figure 1:**
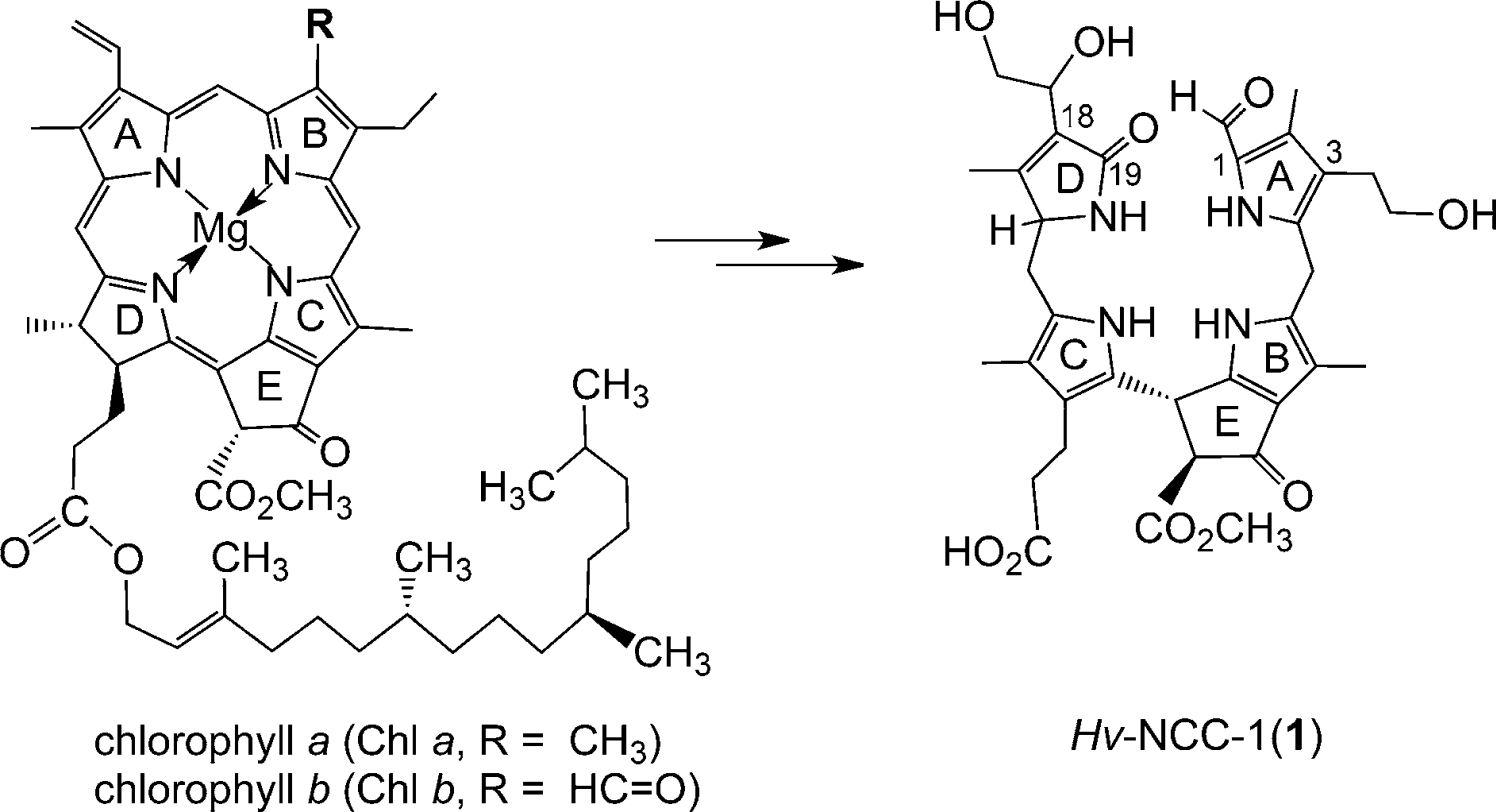
“Rusty pigment 14” from senescent leaves of barley (*Hordeum vulgare*), later named *Hv*‐NCC‐1 (**1**), was identified as the first nongreen Chl catabolite.8a

**Figure 2:**
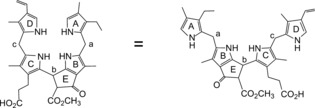
Phyllobilane (**I**),^10^ the name‐giving structure, depicted in two representative formulas to highlight its pseudocyclic (left) and extended conformations (right).

The polar linear tetrapyrrole **1** from barley (*H. vulgare*) was classified as a nonfluorescent Chl catabolite (NCC), and provisionally named *Hv*‐NCC‐1.9a, 18 Its detailed structure analysis established **1** as a 3^2^,18^1^,18^2^‐trihydroxy‐16,19‐dihydro‐1‐formyl‐19‐oxophyllobilane (Figures 1 and 2),^8^, ^19^ named on the basis of a semisystematic, structure‐based nomenclature, according to which linear tetrapyrrolic Chl catabolites are phyllobilin derivatives.9c, 10 The “phyllobilin” terminus refers to the basic bilin‐type structures of such Chl catabolites, and to chlorophyll as their origin. The name‐giving compound is the phyllobilane (**I**, Figure 2). In analogy to the nomenclature of linear tetrapyrroles and bile pigments,^3^ NCCs with three saturated *meso* positions are, hence, classified as 16,19‐dihydro‐1‐formyl‐19‐oxophyllobilanes, such as, for example, the 16,19‐dihydro‐1‐formyl‐19‐oxo‐16‐*epi*‐phyllobilane (***epi*** **‐5**), identified in senescent leaves of the Katsura tree (*Cercidiphyllum japonicum*; see Table 1 in Section 4.1).^20^

Structure elucidation of 1‐formyl‐19‐oxophyllobilane **1** provided a first firm central focal point for considerations on the enigmatic pathway of Chl breakdown during leaf senescence:8a The chemical constitution of **1** indicated loss of the central magnesium ion and of the phytyl group during breakdown of Chl. The structure of NCC **1** also implied an oxygenolytic opening of the porphyrinoid macrocycle at the northern *meso* position, thereby revealing a regioselectivity of Chl breakdown that was completely unexpected on the basis of model reactions with Chl derivatives.4b It was reminiscent, in a striking way, of the breakdown of heme,^3^ in which the macrocycle of heme is also opened at the analogous α‐*meso* position. However, in contrast to oxidation of the methine unit (of Chl) to the formyl group that is characteristic of NCC **1**, typical heme catabolites are 1,19‐dioxobilins, which lack the carbon atom at the site of the cleavage of the macrocycle (which is removed as carbon monoxide by a reaction catalyzed by the heme oxygenase).^21^ As **1** carried a methyl group at its 2‐position, it also appeared to be more closely related to Chl *a* than to Chl *b*.8a, 22 However, compared to Chl *a*, additional polar functional units were attached at the periphery of **1**, thus rendering it rather soluble in water. Furthermore, the bilane‐like **1** was indicated to exist in a variety of stable conformations with respect to its three saturated *meso* positions.

### 1.2 Nonfluorescent Chl Catabolites Accumulate in Senescent Leaves

**Figure 3:**
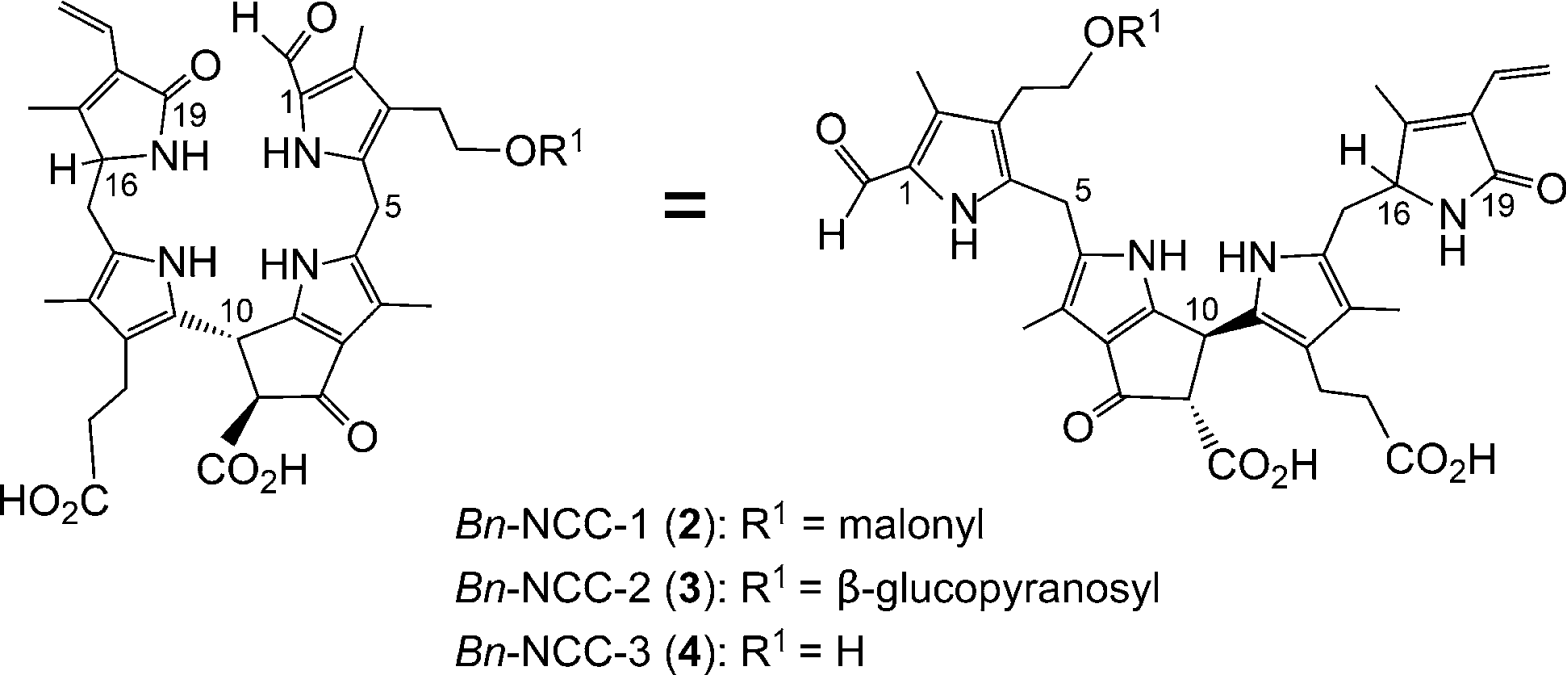
NCCs detected in naturally senescent cotyledons of *Brassica napus* (*Bn*‐NCCs **2**–**4**)^23^ depicted in representative formulas to highlight its pseudocyclic (left) and extended conformations (right).

The initially identified *Hv*‐NCC‐1 (**1**) was obtained from leaves that degreened upon storage in the dark. The use of this conventional method of artificially inducing leaf senescence raised the question of the more general validity of the surprising structure of the NCC **1**. Fortunately, *Bn*‐NCC‐1 (**2**), *Bn*‐NCC‐2 (**3**), and *Bn*‐NCC‐3 (**4**; Figure 3), related polar NCCs in naturally senescent leaves of oilseed rape (*Brassica napus*), were soon identified, thereby substantiating the role of NCCs as colorless products of natural Chl breakdown.18a, 23

## 2 Chlorophyll Breakdown—a Cellular Three‐Compartment Pathway

**Figure 4:**
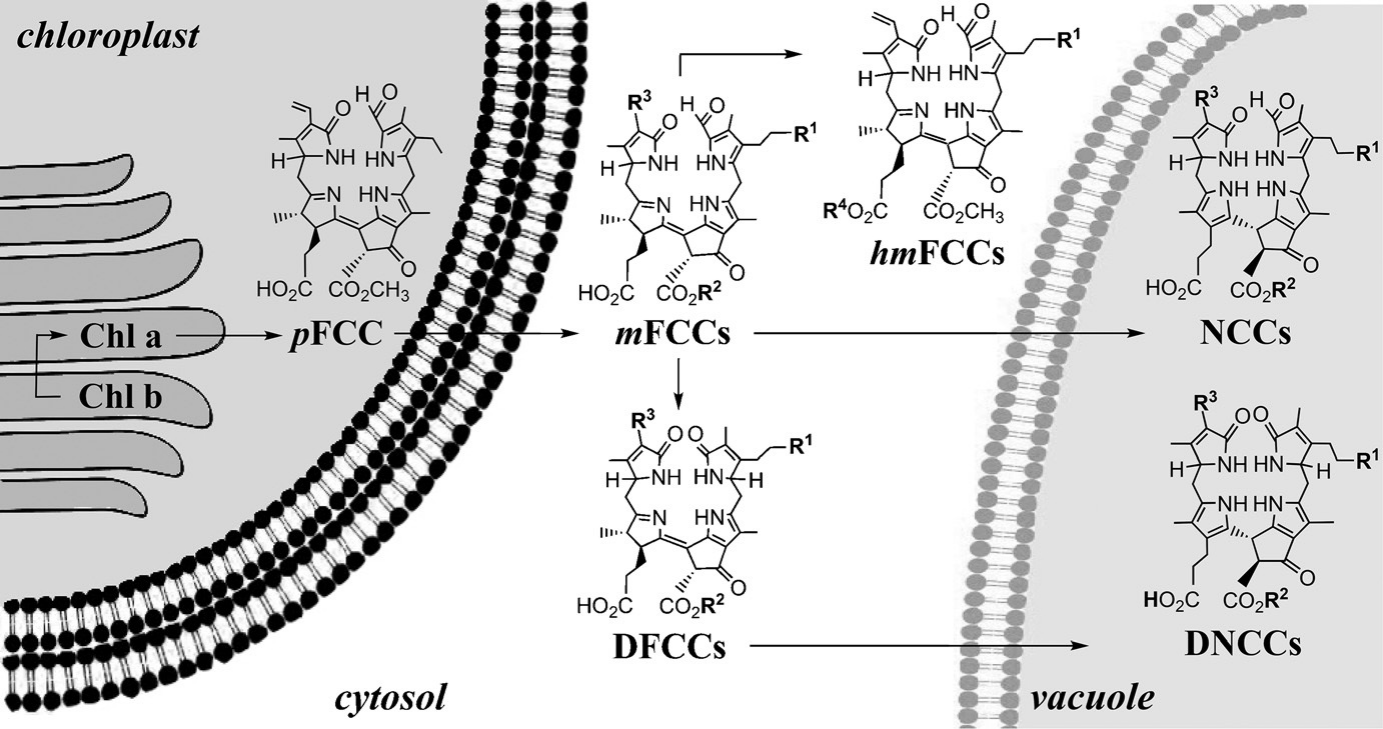
A simplified topographical model of Chl breakdown in a leaf cell, with relevant organelles and key types of Chl catabolites depicted.

Matile et al. found evidence for the localization of NCCs in the vacuoles.13c, 24 Thus, catabolites of Chl, which originate in the chloroplasts, would need to pass through the cytosol to gain access to the vacuoles.9c, 24 The breakdown of Chl was, therefore, proposed to involve metabolic processes in the three main compartments of leaf cells, including (unidirectional) transport between them.1a, 9a Subsequent research indicated an original fluorescent Chl catabolite (**6**, an FCC or 1‐formyl‐19‐oxophyllobilene‐*b*)^25^ as a colorless product of Chl breakdown in the chloroplasts. FCCs (similar to **6**) were deduced to be exported into the cytosol, where further modified FCCs (*m*FCCs) would be generated.9c Subsequently, typical *m*FCCs would be transported into the vacuoles to undergo rapid isomerization to the corresponding polar NCCs, lose their characteristic formyl group to furnish dioxobilin‐type fluorescent Chl catabolites (DFCCs,^26^ the precursors of corresponding dioxobilin‐type nonfluorescent Chl catabolites, DNCCs),^26^, ^27^ or become persistent hypermodified FCCs (*hm*FCCs) that accumulate in leaves and fruit (and give the ripe bananas their blue glow; Figure 4).6b, 28 Thus, a quarter of a century of collaborative chemical and biological research on Chl breakdown has not only revealed an unexpected structural variety of different natural Chl catabolites, but also key enzymes involved in the formation of the catabolites (see Sections 3 and 4).1a, 8a, 9a,9c, 10

## 3 Common Early Part of the PaO/Phyllobilin Pathway

### 3.1 From Chlorophylls a and b to Pheophorbide a

The apparent close structural relationship of NCCs to Chl *a* raised the question of the whereabouts of the remains from the breakdown of Chl *b*. The observed specificity of the ring cleavage reaction for pheophorbide *a* (Pheo *a*) during Chl breakdown underlined this problem.^29^ However, isotopic labeling experiments by Folley and Engel with *Hv*‐NCC‐1 (**1**) showed significant incorporation of deuterium into the C2‐methyl group of **1**, thus supporting the origin of **1** from the degradation of both Chl *a* and Chl *b*, and suggesting the relevance of a reduction of the C2‐formyl group of Chl *b*.^30^ Chl *a*/*b* interconversions were, indeed, revealed not only for the biosynthetic oxidative branch from chlorophyllide *a* to chlorophyllide *b*,^31^ but also its surprising reductive catabolic counterpart that converts Chl *b* back into Chl *a*, and involves 7^1^‐hydroxy‐Chl *a* as an intermediate.^32^ In this way, a so‐called Chl cycle11b, 33 was shown to regulate the relative levels of Chl *a* and Chl *b* throughout the development of the plant, as well as in the initial stages of Chl breakdown in senescent plant tissues.9c, 32

**Figure 5:**
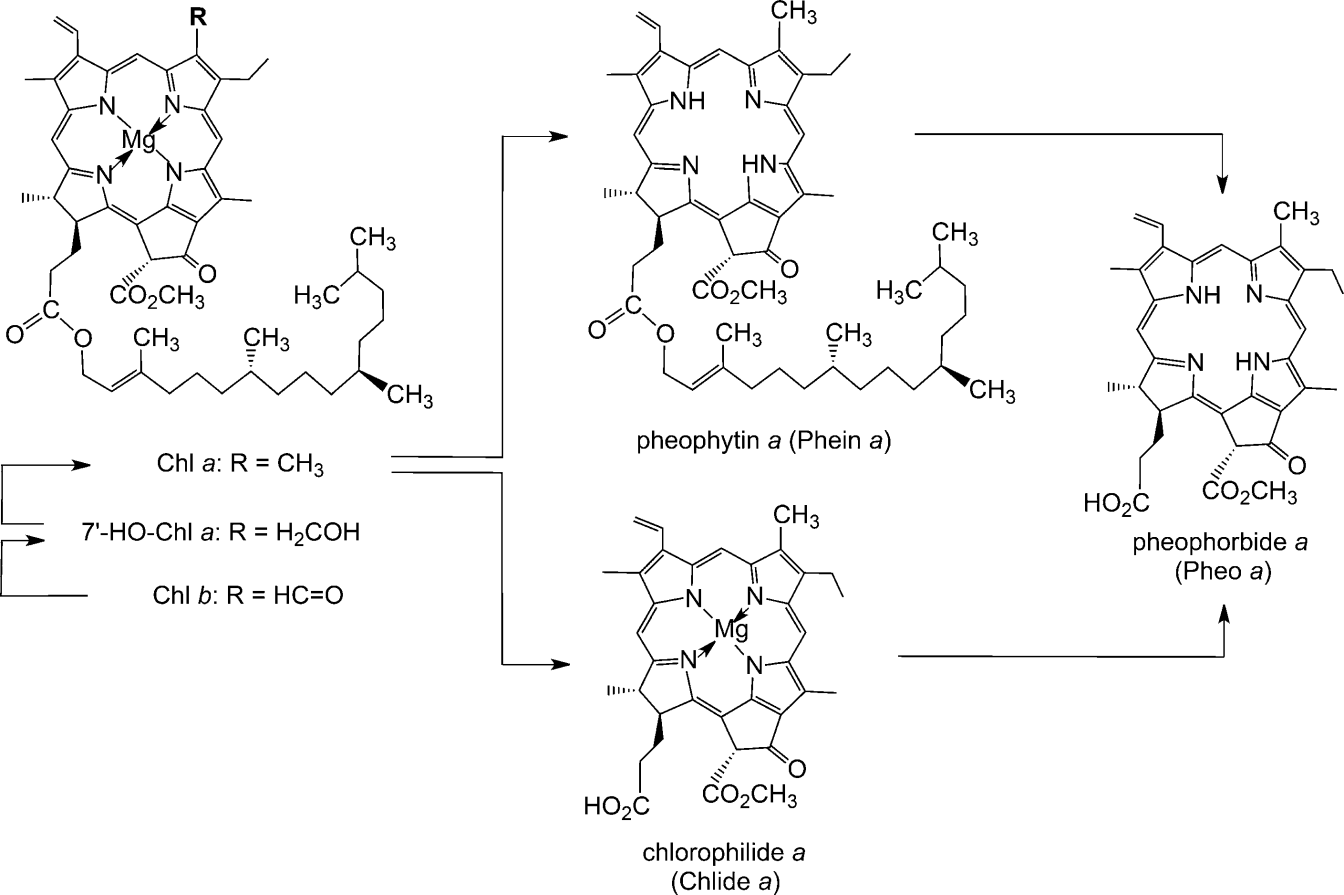
Early steps of Chl breakdown in the chloroplasts produce pheophorbide *a* (Pheo *a*).

Surprisingly, two different pathways are available in higher plants for the degradation of Chl *a* to Pheo *a* (Figure 5).9c One of these, relevant in ripening citrus fruits, involves ester hydrolysis by chlorophyllase^34^ to give chlorophyllide *a* (Chlide *a*). This is followed by removal of the Mg ion to furnish Pheo *a*.^35^ However, this sequence is not functional in some senescent leaves, in which pheophytin *a* (Phein *a*), the suggested product of direct Mg‐removal from Chl *a*, was observed as an intermediate of Chl breakdown.^36^ In *A. thaliana*, the serine‐type hydrolase pheophytinase was identified recently, which converts Phein *a* into Pheo *a*, but does not hydrolyse Chl *a*.^37^ Thus, in senescent leaves of several plants, in vivo removal of the Mg ion from Chl *a* occurs first, followed by hydrolysis of the phytyl ester.9c

### 3.2 The Red Chlorophyll Catabolite—The Native Phyllobilin

The intriguing structures of NCCs (1‐formyl‐19‐oxo‐phyllobilanes) as late breakdown products called for the identification of earlier intermediates of Chl degradation.^38^ Lipofuscin‐like fluorescing compounds that could be identified in extracts of senescent leaves due to their blue fluorescence were suspected to be intermediate products of Chl breakdown.^39^ The blue‐fluorescing compound **6** (originally named *Bn*‐FCC‐2) was first prepared from Pheo *a* by the use of an enzyme‐active extract of senescent leaves of oilseed rape (*Brassica napus*).^25^ Its molecular formula (C_35_H_40_N_4_O_7_)^40^ confirmed a close relationship to Pheo *a*, with net addition of two O atoms and of four H atoms. Blue‐fluorescing *Bn*‐FCC‐2 (**6**) was, thus, proposed to be an intermediate of Chl breakdown and suggested to represent a product of Pheo *a* oxygenolysis.^25^ Pheo *a*, indeed, appeared to be degraded in a process requiring molecular oxygen.^29^ Furthermore, since Pheo *a* accumulated in leaves in the absence of oxygen, but not Pheo *b*, Pheo *a* was considered the last green intermediate of Chl breakdown in senescent leaves.^29^ This role of Pheo *a* required reduction of Chl *b* to Chl *a* as an early step of Chl breakdown.^31^, ^33^

**Figure 6:**
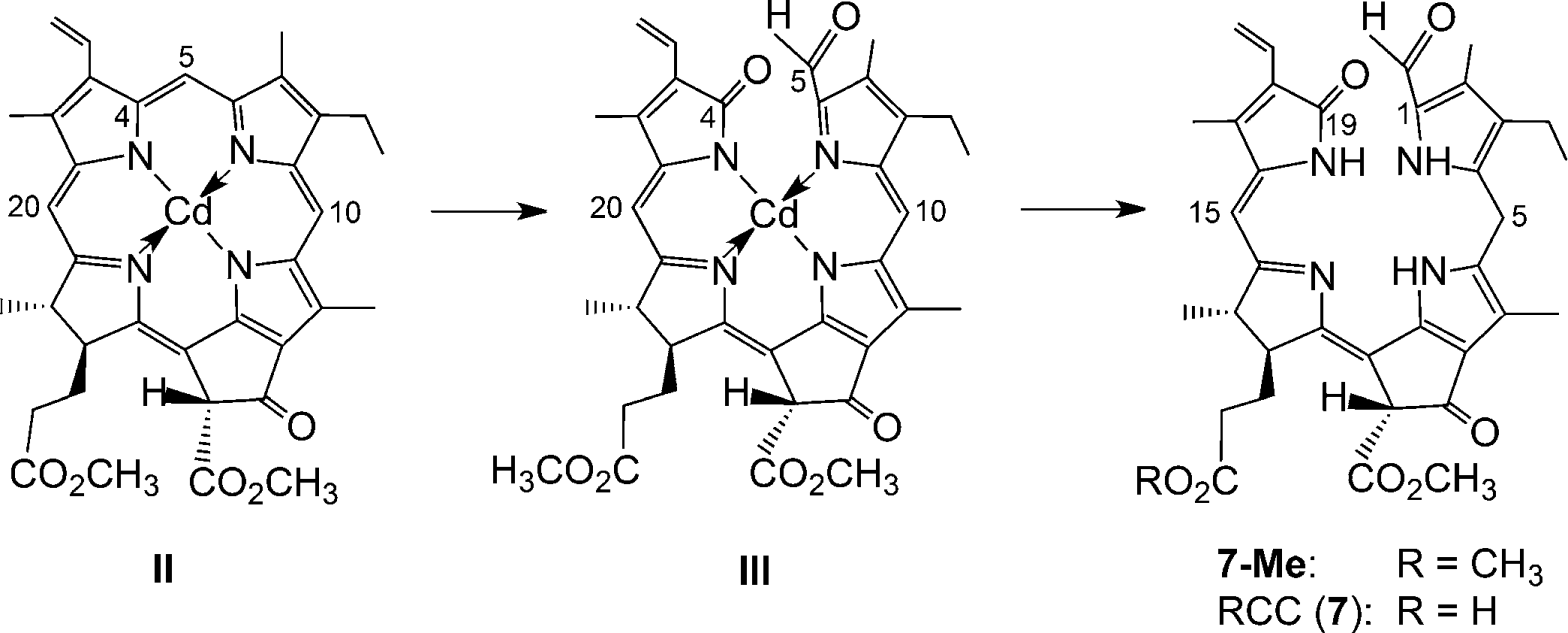
Photooxidation of the Cd^II^‐pheophorbidate **II** and reduction of the 4,5‐dioxosecophytoporphyrin **III** furnishes RCC (**7**) via its methyl ester precursor **7‐Me**.^42^

**Figure 7:**
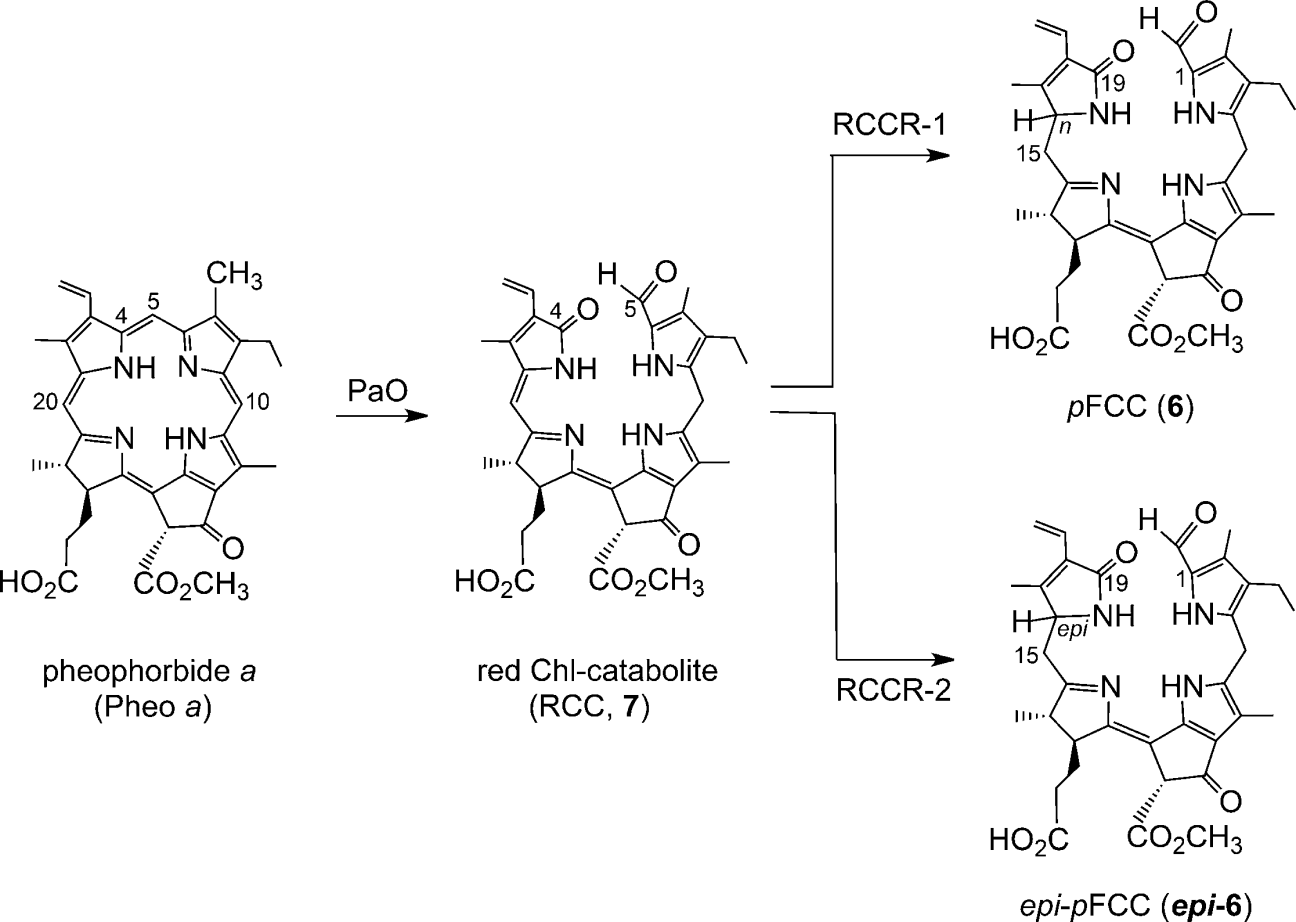
Chl breakdown from Pheo *a* via RCC (**7**) to primary FCCs (**6**/***epi*** **‐6**) is catalyzed by Pheo a oxygenase (PaO) and by two classes of RCC reductases (RCCR‐1 and RCCR‐2).9c

What would be a rational product of the oxygen‐dependent degradation of Pheo *a* and how could it relate to blue‐fluorescent **6**?^25^ Despite the documented failures of detecting colored remains of Chl in natural plant sources, the unknown red bilin‐type catabolite **7** (Figures 6 and 7) appeared to represent such a rational hypothetical precursor of **6**.^25^ Studies by Gossauer and Engel^41^ on Chl catabolites in *Auxenochlorella protothecoides* (earlier named *Chlorella protothecoides*) had, indeed, revealed the existence of similar red, Chl‐derived linear tetrapyrroles in secretions of this green alga.

It became crucial to test the role of such a hypothetical red intermediate in an early phase of Chl catabolism. Thus, the red 12,13‐dihydro‐1‐formyl‐19‐oxophyllobiladiene‐*b*,*c* **7** (now commonly called red Chl catabolite, RCC) was prepared from Pheo *a* by partial chemical synthesis.^42^ In analogy to experiments by the Gossauer research group with Cd^II^ complexes of pyropheophorbide *a* (13^2^‐desmethoxycarbonyl‐Pheo *a*),^43^ the Cd^II^ complex of Pheo *a* methyl ester (**II**) was photooxidized to furnish Cd^II^‐[4,5]‐dioxo‐[4,5]‐seco‐4,5‐dihydromethylpheophorbidate **III** in about 30 % yield (Figure 6). The brownish and rather unstable oxidation product was readily reduced with NaBH_4_ to give the deep‐red RCC methyl ester (**7‐Me**).^42^ Enzymatic hydrolysis of **7‐Me** with porcine liver esterase selectively generated a first sample of authentic RCC (**7**) nearly quantitatively.^42^

When synthetic **7** was available as a reference, traces of the very same (previously elusive) red compound were detected when Pheo *a* was incubated with extracts of chloroplasts of senescent *Brassica napus* cotyledons.^44^ Indeed, RCC was eventually revealed as the product of a Rieske‐type monooxygenase, named Pheo *a* oxygenase (PaO),^45^ that uses Pheo *a* selectively as its substrate and that is inhibited by Pheo *b*.^46^ Therefore, RCC (**7**) carries the hallmarks of the oxygenolytic ring opening by PaO, considered the key step of the PaO/phyllobilin pathway of Chl breakdown (Figure 7).9c, 29, 46 In this enzyme‐catalyzed process, the chlorin‐type macroring of Pheo *a* is cut open between C4 and C5, and one oxygen atom is specifically incorporated into the newly produced, characteristic formyl group of (an enzyme‐bound form of) RCC (**7**).^46^ Thus, RCC (**7**) is the native 1‐formyl‐19‐oxobilin‐type Chl catabolite, or native phyllobilin.

### 3.3 Primary Fluorescent Chl Catabolites—Epimeric Pair of Phyllobilin Ancestors

**Figure 8:**
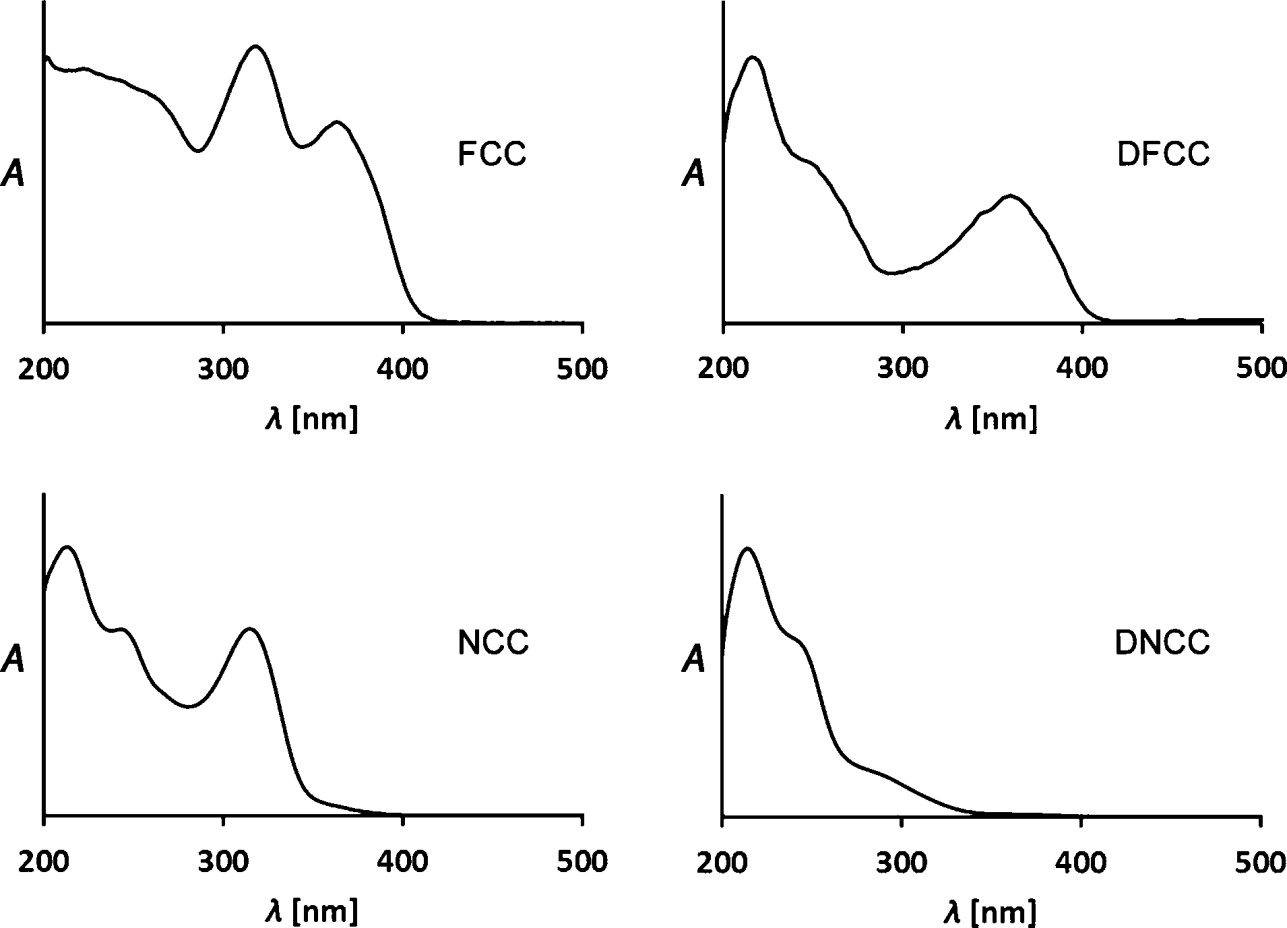
UV spectra of representative colorless phyllobilins of type‐I (FCC and NCC) and of type‐II (DFCC and DNCC).26a

Synthetic RCC (**7**) was shown in extracts of senescent *B. napus* leaves to be reduced to the presumed Chl catabolite **6**, provisionally called *Bn*‐FCC‐2.^44^ NMR spectroscopic studies^17^ had already shown that *Bn*‐FCC‐2 (**6**) was a 12,13,16,19‐tetrahydro‐1‐formyl‐19‐oxophyllobilene‐*b* (Figure 7). As **6** exhibited the same pattern of peripheral functional groups as Pheo *a*, and was not modified further by polar groups known from the NCC structures, it was called the primary FCC (*p*FCC).^25^ In addition to the characteristic absorption maximum of an α‐formylpyrrole unit (ring A) near *λ*=315 nm,8a the UV/Vis spectra of **6** showed a new maximum at *λ*=360 nm, corresponding to a new chromophore extending over rings B and C (Figure 8).6b, 25 In contrast to the nonluminescent NCCs, solutions of FCC **6** exhibit a strong and characteristic blue fluorescence, with an emission maximum near *λ*=450 nm. It was this feature that led to the original phenomenological classification as an FCC.6b, 25

By using an aerated enzyme assay based on a suspension of chromoplasts of red sweet pepper (*Capsicum annum*), Pheo *a* was transformed into another blue‐fluorescing compound, originally called *Ca*‐FCC‐2, and identified as an isomer of *p*FCC (**6**).^47^ Detailed NMR‐spectroscopic analysis indicated a configurational difference between **6** and *Ca*‐FCC‐2 at C16 (C1 in the earlier phytoporphyrin‐based nomenclature^47^). *Ca*‐FCC‐2 was, therefore, named *epi*‐*p*FCC (***epi*** **‐6**, Figure 7).^47^

Reduction of RCC (**7**) to *p*FCC (**6**) was shown to be accomplished by a cofactor‐free RCC reductase (RCCR).^48^ The identification of **6** and its C16 epimer ***epi*** **‐6** suggested two stereospecific classes of RCCRs in higher plants. This conclusion was verified by studies of the RCCRs from a range of plants and their classification as RCCR‐1 and RCCR‐2.^49^ RCCR of *B. napus* is a RCCR‐1 and achieves a highly stereo‐ and regioselective reduction of RCC (**7**) to *p*FCC (**6**).^25^, ^48^ The same (regio‐ and) stereoselectivity is deduced for RCCRs from some other plants, among them *A. thaliana*.49b In contrast, in a second group of plants, among them spinach (*Sp. oleracea*)^50^ and bananas (*M. acuminata*),^51^ C16‐epimeric NCCs were found as descendants of *epi‐p*FCC (***epi*** **‐6**) from the reduction of RCC (**7**) by an enzyme of the RCCR‐2 class.49a RCC reductase (RCCR) is a constitutively expressed enzyme, now classified as belonging to the family of the (ferredoxin‐dependent) bilin reductases.^52^ The crystal structure of RCCR from *A. thaliana* was analyzed in substrate‐free and RCC‐loaded forms (see Refs. 53, 54 for structural details).

### 3.4 Partial Synthesis of pFCC and epi‐pFCC—A Chemical Interlude

**Figure 9:**
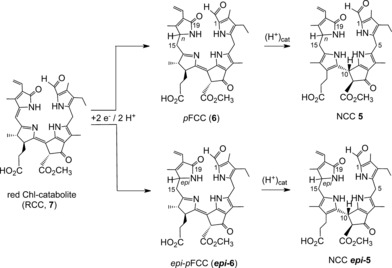
Bio‐inspired partial chemical synthesis of the NCCs **5** and ***epi*** **‐5** by electrochemical reduction of RCC (**7**) to *p*FCC and *epi‐p*FCC (**6** and ***epi*** **‐6**), followed by stereoselective acid‐catalyzed isomerization to **5** and ***epi*** **‐5**.20b

Controlled‐potential electrolytic reduction of RCC methyl ester (**7‐Me**) in a protic solvent system furnished FCC methyl esters **6‐Me** and ***epi*** **‐6‐Me** in about 25 % yield, and with negligible stereoselectivity.^55^ This (chemical) reaction represented a first model process for the reaction catalyzed by the cofactor‐free RCCRs, exploring the ease of the one‐electron reduction of **7‐Me**. Likewise, *p*FCC (**6**) and *epi‐p*FCC (***epi*** **‐6**) were similarly obtained by electrochemical reduction of synthetic RCC (**7**), and revealed useful regioselectivity for the addition of hydrogens at the 15‐ and 16‐positions, but an absence of significant stereoselectivity (Figure 9).20b Hence, this experiment provided access to both C16 epimers of the natural primary FCCs.

The facile electrochemical reduction of **7** to **6** and ***epi*** **‐6** in weakly acidic solution was in line with a ferredoxin‐driven reduction^54^, ^56^ catalyzed by RCCRs. Indeed, enzymatic reduction of RCC (**7**) by RCCR was deduced to follow a related mechanistic sequence, through protonation and single electron transfer reduction steps.^54^

## 4 Branching of the PaO/Phyllobilin Pathway in its Later Stages

**Figure 10:**
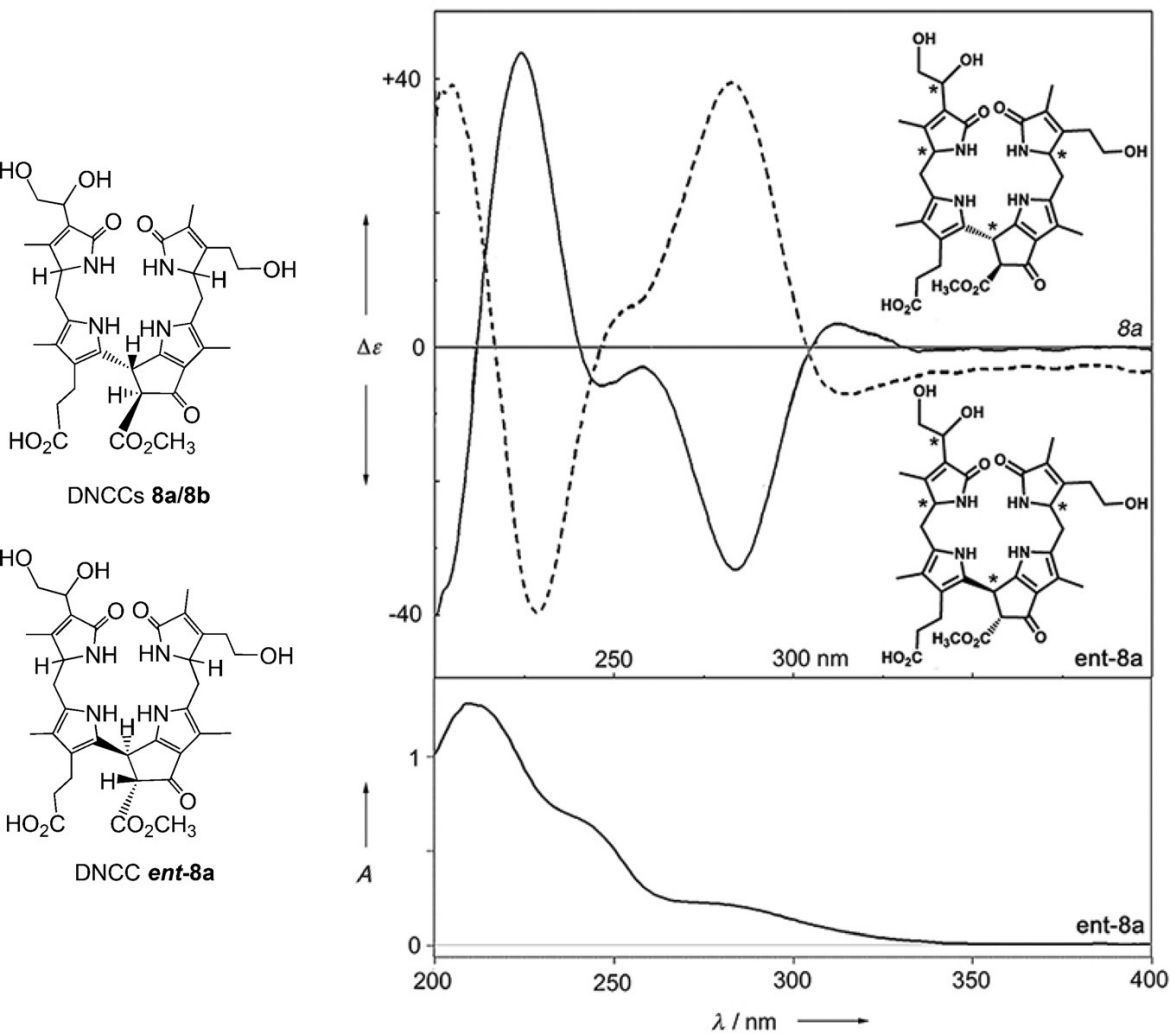
Structural formulas (left) of DNCCs **8 a**/**8 b** (from barley)^57^ and ***ent*** **‐8 a** (from a Norway maple leaf),^58^ whose CD and UV spectra (right) feature the properties of an intriguing enantiomer of **8 a**.

Degreened leaves of barley (*H. vulgare*) were not only the first source of the colorless NCC **1**, but also of a second major type of nonfluorescent and colorless Chl catabolites, the urobilinogenoidic Chl catabolites (UCCs) **8 a** and **8 b**.^57^ The two epimers, **8 a** and **8 b**, found in the barley leaf extracts, appeared to be direct descendants of *Hv*‐NCC‐1 (**1**).^57^ However, consistent with the absence of a formyl group, the UV spectra of these phyllobilins lacked the absorption band near *λ*=315 nm, which is typical of NCCs, such as **1**. The tetrapyrroles **8 a** and **8 b** are now classified, in a structure‐based way, as 1,19‐dioxobilin‐type NCCs (DNCCs, Figure 10).^10^ More recently, the major colorless phyllobilin in senescent leaves of Norway Maple (*Acer platanoides*) was also characterized as a DNCC, and shown by its CD spectra to behave as the enantiomer (***ent*** **‐8 a**) of the barley DNCC **8 a**.^58^ This striking finding led to the suggestion that DNCCs may not be derived from NCCs, but would probably originate from an earlier Chl‐breakdown intermediate, thus basically indicating a divergent pathway of Chl breakdown (see Section 4.2).^58^

1‐Formyl‐19‐oxobilin‐type Chl catabolites only appear to be present in a variety of senescent leaves and ripe fruit, such as spinach leaves,^50^ apples, and pears.^59^ In striking contrast, only the remarkable 1,19‐dioxobilin‐type Chl catabolite ***ent*** **‐8 a** was detected in senescent leaves of Norway maple.^58^ However, both lines of colorless phyllobilins were present in senescent leaves of *Arabidopsis thaliana* 27, 60 and in broccoli florets (*B. oleracea*).^61^

**Figure 11:**
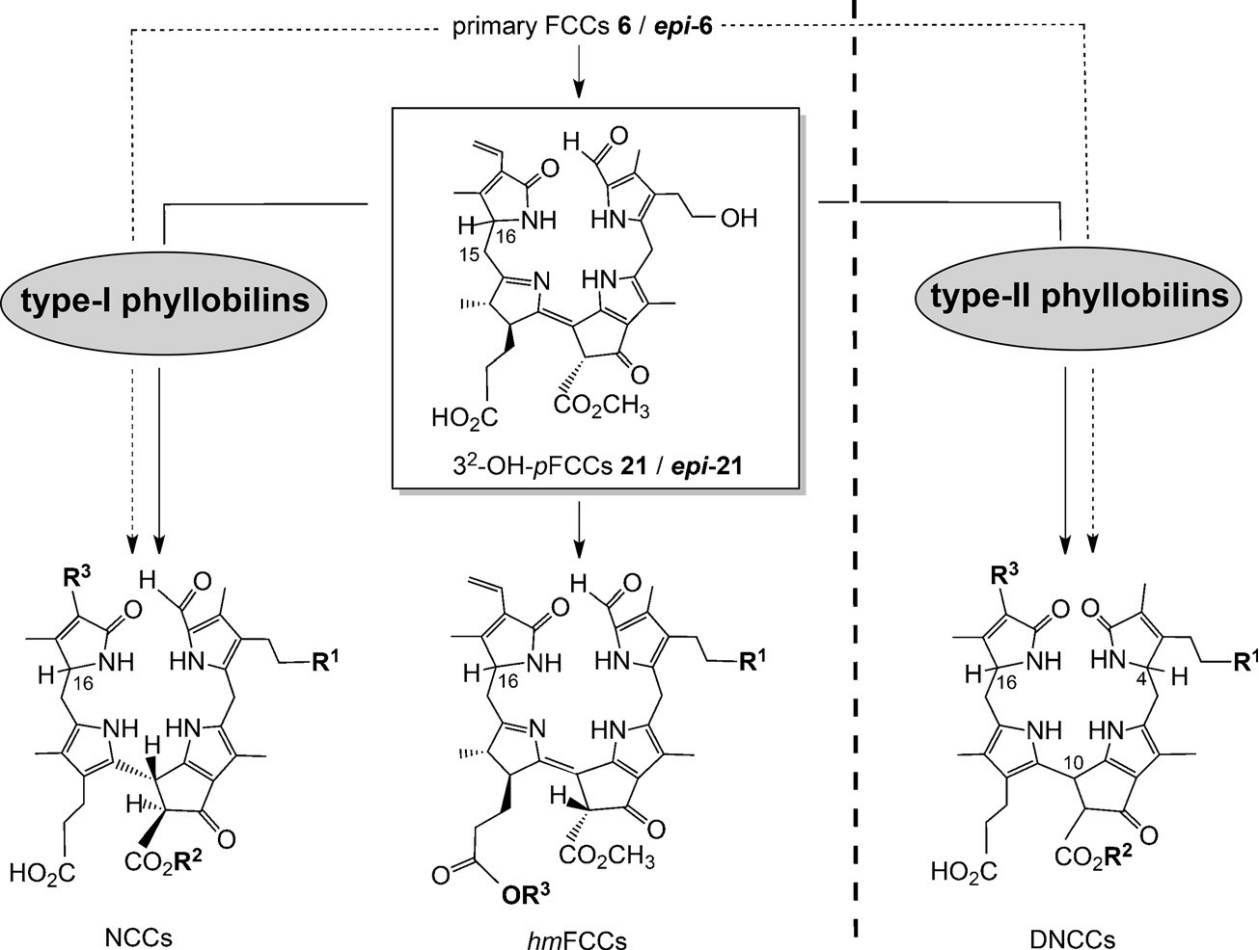
Branching of Chl breakdown occurs at the level of FCCs and provides pathways to downstream type‐I and type‐II phyllobilins (see Tables 1–3 for examples of R^1^, R^2^, and R^3^).^10^

As we now know, 1,19‐dioxobilin‐type Chl catabolites (DCCs), which have in the meantime been classified as type‐II phyllobilins, branch off from the original lineage of the 1‐formyl‐19‐oxophyllobilins, or type‐I phyllobilins. Branching occurs at the stage of FCCs, where oxidative deformylation competes with pathways to other downstream type‐I phyllobilins (*hm*FCCs, NCCs) and leads mainly to 1,19‐dioxobilin‐type NCCs (DNCCs).^10^, 26a Two such branching points have, so far, been identified in *A. thaliana* (Figure 11).26c

### 4.1 The Original Type‐I Phyllobilins—Colorless 1‐Formyl‐19‐oxobilins

**Figure 12:**
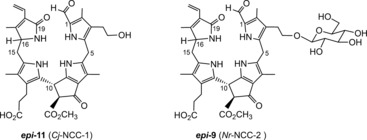
Constitutional formulas of two common natural NCCs. Catabolites ***epi*** **‐11** and ***epi*** **‐9** (first identified as *Cj*‐NCC‐1 and *Nr*‐NCC‐2, respectively), which are also present in the peels of apples and pears (see Table 1).

Over the years, about 20 structurally different natural (nonfluorescent) 1‐formyl‐19‐oxophyllobilanes (NCCs or type‐I phylloleucobilins) have been characterized, firmly establishing their broad relevance as final Chl catabolites in senescent leaves of various plants. Among these were, for example, the NCCs **1**–**4** (see Sections 1 and 2), ***epi*** **‐9** and ***epi*** **‐10** from tobacco (*Nicotiana rustica* 62), ***epi*** **‐5** and ***epi*** **‐11** from *C. japonicum* 20a, 63), ***epi*** **‐1**, ***epi*** **‐5**, ***epi*** **‐11**, ***epi*** **‐12**, and ***epi***‐**13** from spinach^50^, ^64^ (*Spinacia oleracea*), ***epi*** **‐9** and ***epi*** **‐14** from maize^65^ (*Zea mais*), as well as **3**, **4**, **15**–**17** from *A. thaliana* 60, 66 (see Table 1 and Figure 12).^10^ NCCs were identified tentatively in extracts of senescent leaves on the basis of their characteristic UV‐absorption maximum near *λ*=315 nm (Figure 8).^8^ ESI‐MS spectra helped to establish the molecular formula. Characteristic fragments often allowed conclusions concerning functional groups.16c The ^1^H NMR spectra of NCCs show a characteristic singlet corresponding to the CH=O group at low field.8a The constitution of the phyllobilins and part of their relative configuration was deduced from analysis of high‐field ^1^H,^1^H homonuclear and ^1^H,^13^C heteronuclear NMR spectra.8a, 10, 17a

**Table 1:** Structures of natural 1‐formyl‐19‐oxophyllobilanes or nonfluorescent Chl catabolites (NCCs).

No.^[a]^	R^1[b]^	R^2[b]^	R^3[b]^	C16^[c]^	Original name (identical with)^[d]^	Ref.
**17**	H	H	CH=CH_2_	n	*Bo*‐NCC‐2 (*At*‐NCC‐5/*Bn*‐NCC‐4^[e]^)	60, 61
**15**	H	H	CH=CH_2_	n	*At*‐NCC‐3^[f]^	66
***epi*** **‐5**	H	CH_3_	CH=CH_2_	epi	*Cj*‐NCC‐2 (*So*‐NCC‐5)	20a,20b, 50
**4**	OH	H	CH=CH_2_	n	*Bn*‐NCC‐3 (*At*‐NCC‐2^[e]^)	23, 60
***epi*** **‐13**	OH	H	CH=CH_2_	epi	*So*‐NCC‐3 (*Mc*‐NCC‐49/*Ej*‐NCC‐3^[e]^)	50, 51, 83
***epi*** **‐12**	OH	H	CH(OH)‐CH_2_OH	epi	*So*‐NCC‐1 (*Mc*‐NCC‐26)	50, 51
**11**	OH	CH_3_	CH=CH_2_	n	*Sw*‐NCC‐58	91
***epi*** **‐11**	OH	CH_3_	CH=CH_2_	epi	*Cj*‐NCC‐1 (*So*‐NCC‐4/*Pc*‐NCC‐2/*Md*‐NCC‐2/	50, 51, 59, 63
					*Mc*‐NCC‐61/*Ej*‐NCC‐4^[e]^)	83
**1**	OH	CH_3_	CH(OH)‐CH_2_OH	n	*Hv*‐NCC‐1	8
***epi*** **‐1**	OH	CH_3_	CH(OH)‐CH_2_OH	epi	*So*‐NCC‐2 (*Mc*‐NCC‐42/*Ej*‐NCC‐1^[e]^)	50, 51, 64, 83
**3**	O‐Glc	H	CH=CH_2_	n	*Bn*‐NCC‐2 (*At*‐NCC‐1^[e]^/ *Bo*‐NCC‐1)	23, 60, 61
**16**	O‐Glc	CH_3_	CH=CH_2_	n	*At‐*NCC‐4^[e]^	60
***epi*** **‐9**	O‐Glc	CH_3_	CH=CH_2_	epi	*Nr*‐NCC‐2 (*Zm*‐NCC‐2/*Pc*‐NCC‐1/*Md*‐NCC‐1/ *Tc*‐NCC‐2/*Mc*‐NCC‐59)	51, 59, 62, 65, 92
***epi*** **‐14**	O‐Glc	CH_3_	CH(OH)‐CH_2_OH	epi	*Zm*‐NCC‐1 (*Tc*‐NCC‐1/*Co*‐NCC‐1^[e]^)	65, 82, 92
***epi*** **‐18**	O‐Glc	CH_3_	CH(OH)‐CH_2_O‐Glc	epi	*Pd‐*NCC‐32	68
***epi*** **‐10**	O‐MalGlc	CH_3_	CH=CH_2_	epi	*Nr*‐NCC‐1	62
**2**	O‐Mal	H	CH=CH_2_	n	*Bn*‐NCC‐1	18a, 23
***epi*** **‐29**	O‐Mal	CH_3_	CH=CH_2_	epi	*Ej*‐NCC‐2^[e]^	83
***epi*** **‐20**	OH	CH_3_	CH=CH_2_	epi	*M*c‐NCC‐58^[g,h]^	51
***epi*** **‐21**	OH	CH_3_	CH=CH_2_	epi	*Mc*‐NCC‐55^[h,i]^	51

**Figure 13:**
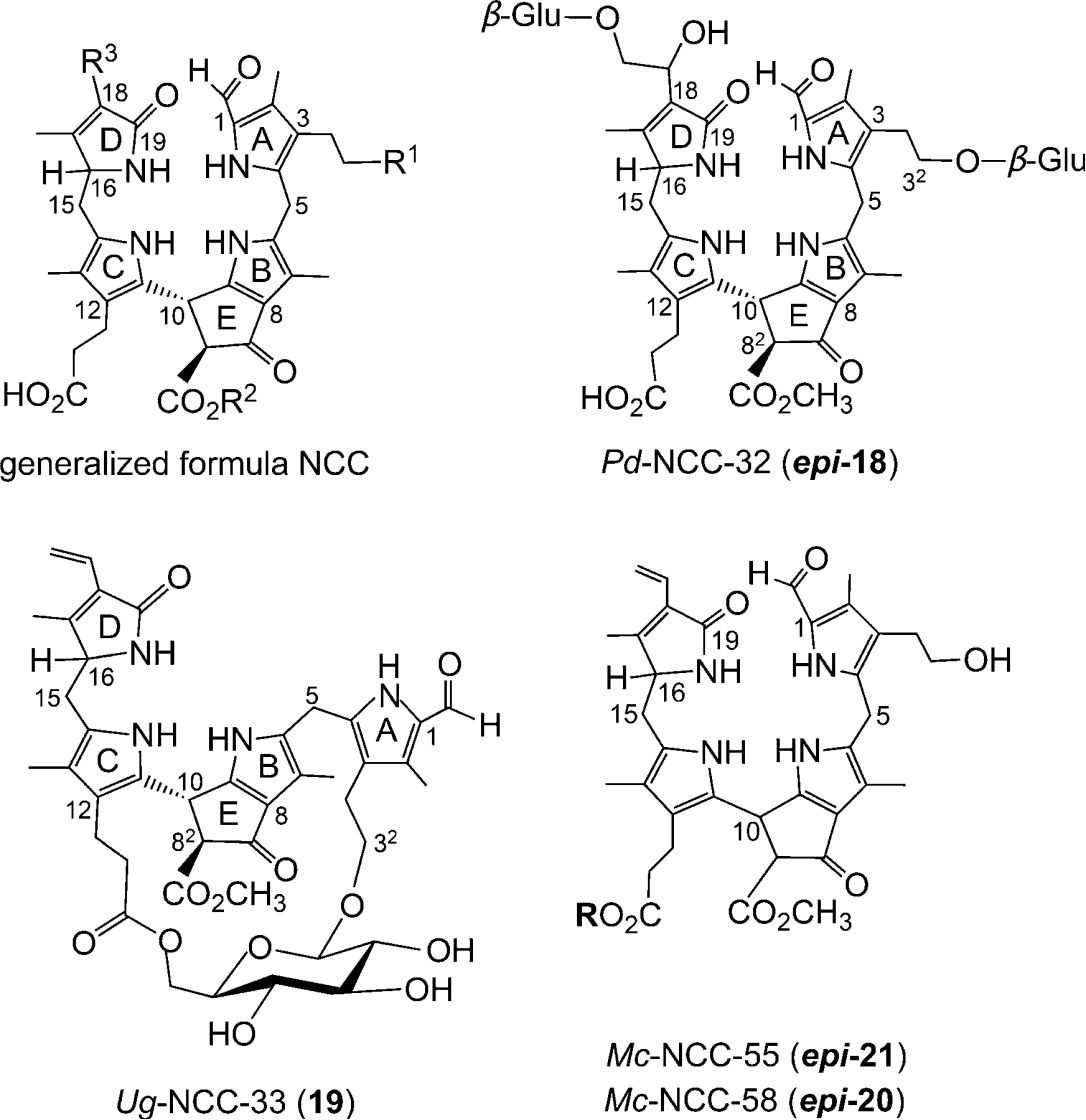
Generalized NCC formula, including atom numbering (top left) and structural formulas of natural NCCs with unique structures: doubly glycosylated *Pd*‐NCC‐32 (***epi*** **‐18**), bicycloglycosidic *Ug*‐NCC‐3 (**19**), and epimeric NCC esters ***epi*** **‐20**/***epi***‐**21** from banana peels (**R**=daucyl unit, *Mc*‐NCC‐58 has an *R* configuration at C10, *Mc*‐NCC‐55 is the *S* epimer).

[a] Compound number (see text). [b] R^1^ to R^3^ refer to a generalized NCC formula, shown in Figure 13. Abbreviations: Mal=malonyl; Glc=β‐glucopyranosyl, MalGlc=O6′‐(Mal)Glc. [c] The absolute configuration of NCCs at C16 is still unknown; assigned as “n” or as “epi”, when the NCC is derived from *p*FCC (**6**) or from *epi‐p*FCC (***epi*** **‐6**), respectively. [d] *Bo*‐NCCs: *B. oleracea* var. *italica* (broccoli),^61^ *At*‐NCCs: *A. thaliana*,^60^, ^66^ *Bn*‐NCCs: *B. napus* (oilseed rape),18a, 23 *Cj*‐NCCs: *C. japonicum* (Katsura tree),20a, 63 *So*‐NCCs: *Sp. oleracea* (spinach),^50^, ^64^ *Mc*‐NCCs: *M*. *acuminata* (banana peels, Cavendish cultivar),^51^ *Sw*‐NCC‐58: *Sp. wallisii* (Peace Lily),^91^ *Pc*‐NCCs: *P. communis* (pear),^59^ *Md*‐NCCs: *M*. *domestica* (apple),^59^ *Hv*‐NCC‐1, *H. vulgare* (barley),^8^ *Nr*‐NCCs: *N. rustica* (tobacco),^62^ *Zm*‐NCCs: *Z. mays* (maize),^65^ *Tc*‐NCCs: *T. cordata* (lime tree),^92^ *Ej*‐NCCs: *E. japonica* (loquat fruits),^83^ *Co*‐NCCs: *C. oblonga* (quince),^82^ and *Pd*‐NCCs: *P. domestica* (plum tree).^68^ [e] Tentative assignments based on UV/Vis and mass spectra. [f] *At*‐NCC‐3 (**15**) carries a HOCH_2_ group at C2.^66^ [g] *R* Configuration at C10 derived from the CD spectrum.^51^ [h] *S* Configuration at C10 derived from the CD spectrum.^51^ [i] propionate side chain esterified with a daucyl unit.28a, 85

All NCCs (or 16,19‐dihydro‐1‐formyl‐19‐oxophyllobilanes^10^) are flexible linear tetrapyrroles with unconjugated pyrrolic units. As descendants of *p*FCC (**6**) or of *epi‐p*FCC (***epi*** **‐6**), which differ by their absolute configuration at C16 in a species‐specific way, NCCs also occur in two epimeric classes (see, for example, Ref. 64 and Table 1). With one striking exception, NCCs carry a methyl group at C2.^10^ The noted exception is *At*‐NCC‐3 (**15**, from senescent leaves of *A. thaliana*), in which a hydroxymethyl group is attached at C2.^66^ Furthermore, all NCCs, and other known natural phyllobilins from higher plants, feature a methoxycarbonyl group or a carboxy group at their C8^2^‐position (Table 1). This structure of ring E excludes pyropheophorbide *a* (13^2^‐desmethoxycarbonyl‐Pheo *a*) as a rational biological precursor of natural NCCs.

The amphiphilic NCC 3^2^‐hydroxy‐16,19‐dihydro‐1‐formyl‐19‐oxo‐*epi*‐phyllobilane (***epi*** **‐11**) has been encountered in various higher plants,6a for example, as *Cj*‐NCC‐1 in leaves of *C. japonicum*.20a, 63 It carries a hydroxyethyl group at the C3‐position of ring A, which increases its polarity. The phyllobilane ***epi*** **‐11** was also described, for example, as *Pc*‐NCC‐2 and as *Md*‐NCC‐2 in senescent leaves of pear and apple trees, as well as in the corresponding ripe fruit.^59^ Its epimer, the 3^2^‐hydroxy‐16,19‐dihydro‐1‐formyl‐19‐oxophyllobilane **11**, was detected (as *So*‐NCC‐4) in senescent spinach leaves and (as *Sw*‐NCC‐58) in Peace Lily (*Spathiphyllum wallisii*) leaves. The 3^2^‐OH group may serve as an anchor for further attachment of hydrophilic groups at this position. Thus, the 3^2^‐(β‐glucopyranosyl)oxy‐1‐formyl‐19‐oxo‐*epi*‐phyllobilane (***epi*** **‐9**) was discovered in senescent tobacco leaves and originally named *Nr*‐NCC‐2.^62^ Constitutional variability in 1‐formyl‐19‐oxophyllobilanes (or NCCs, see Table 1) was found in the side chains at positions C3, C8, and C18, all resulting from peripheral modifications during catabolism by malonylation,18a methyl ester hydrolysis,^23^, ^67^ and/or dihydroxylation of the C18 vinyl group.8a Unique NCC structures were recently identified in leaves of plum trees (*Prunus domestica*), where *Pd*‐NCC‐32 (***epi*** **‐18**) is glycopyranosylated twice (at O3^3^ and O18^3^),^68^ as well as in leaves of Wich Elm (*Ulmus glabra*), where a bridging glucopyranosyl group generated an unprecedented [17.3.1] bicycloglycosyl motif in *Ug*‐NCC‐3 (**19**; Figure 13).^69^ In addition, traces of other unprecedented, natural NCC esters (*Mc*‐NCC‐58, ***epi*** **‐20** and its C10 epimer *Mc*‐NCC‐55, ***epi*** **‐21**) were identified from peels of ripe bananas by comparison of the HPLC traces with those of authentic reference material obtained from acid‐catalyzed isomerization of *Mc*‐FCC‐56 (see Section 6).^51^ Thus, the NCC structures suggested a range of enzyme‐catalyzed reactions relevant in Chl degradation in senescent leaves, such as hydroxylation, glycosylation, malonylation,^70^ dihydroxylation, methyl ester hydrolysis,^67^ and formation of propionate esters. Hydroxylation at C3^2^ 51 and hydrolysis of the methyl ester function at O8^4^ 60, 67 were deduced to occur with the corresponding FCC precursors. Likewise, glycosylation, malonylation, ester formation, and side chain dihydroxylation, observed in some NCCs, are also expected to involve enzyme‐catalyzed reactions of FCCs. Indeed, modified FCCs (*m*FCCs) with structures reflected by the functional groups observed in NCCs^10^ appear to be functionalized with their respective polar groups before entering the vacuole and undergoing direct isomerization to NCCs there.9c, 20a

The fluorescent Chl catabolites (FCCs or 12,13,16,19‐tetrahydro‐1‐formyl‐19‐oxophyllobilenes‐*b*) and corresponding (nonfluorescent) NCCs (16,19‐dihydro‐1‐formyl‐19‐oxophyllobilanes) are isomers. This structural relationship suggested that FCCs could isomerize into NCCs,20a driven by aromatization of ring C to a pyrrole unit, as seen in hydroporphyrins.^71^ The hypothetical non‐enzymatic isomerization of FCCs to NCCs was tested with both primary FCCs, **6** and ***epi*** **‐6**.20a,20b First, an acid‐induced isomerization of *epi*‐*p*FCC (***epi***‐**6**) was studied, which converted ***epi***‐**6** into the natural *Cj*‐NCC‐2 (***epi*** **‐5**) with high stereoselectivity.20a Isomerization of ***epi*** **‐6** to ***epi*** **‐5** (Figure 9) was rapid at pH 4.9, and ***epi*** **‐6** had a half‐live of less than 30 min.20a Likewise, at pH 4.0, the *p*FCCs **6** and ***epi*** **‐6** isomerized stereoselectively to the respective epimeric NCCs **5** and ***epi*** **‐5** (Figure 9), with apparent first‐order rate constants of 0.020 and 0.039 min^−1^, respectively.6b, 20b

Consistent with their rapid isomerization to NCCs, typical FCCs can only be observed fleetingly in senescent plant material. The free propionic acid group at C12 of ring C activates and steers the (acid‐induced) FCC to NCC isomerization in a remarkably stereoselective way20b (see Ref. 20a for a mechanistic discussion). Indeed, a pH profile of the reaction rate was consistent with the participation of a proton donor with a p*K*_a_ value of about 5, thus supporting the mechanistic view that the isomerization was achieved by an intramolecular protonation of C10 by the propionic acid function. A second carboxylic acid group at the C8^2^‐position of the natural polar *At*‐FCC‐2 (**22**) further accelerated the FCC to NCC isomerization by a factor of about 7 at pH 5 (a probable consequence of a local charge effect).^67^ Therefore, a non‐enzymatic isomerization of FCCs to NCCs was proposed to account for the formation of NCCs in the acidic milieu of the vacuoles, where the free propionic acid function would be partially protonated. The intramolecular protonation step was deduced to preferentially generate NCCs with an *R* configuration at C10. Consistent with the proposed chemical mechanism of the isomerization of natural FCCs to the corresponding NCCs, natural NCCs basically exhibit—with few exceptions—similar CD spectra, thus supporting their common absolute configuration at the (C10) *meso* position between pyrrole rings B and C.20a,20b The deduced *R* configuration of the NCC ***epi*** **‐11** at C10 was recently confirmed by detailed structural analysis, including X‐ray analysis, of a yellow Chl catabolite (YCC) derived from this NCC (see Section 7).^72^

**Figure 14:**
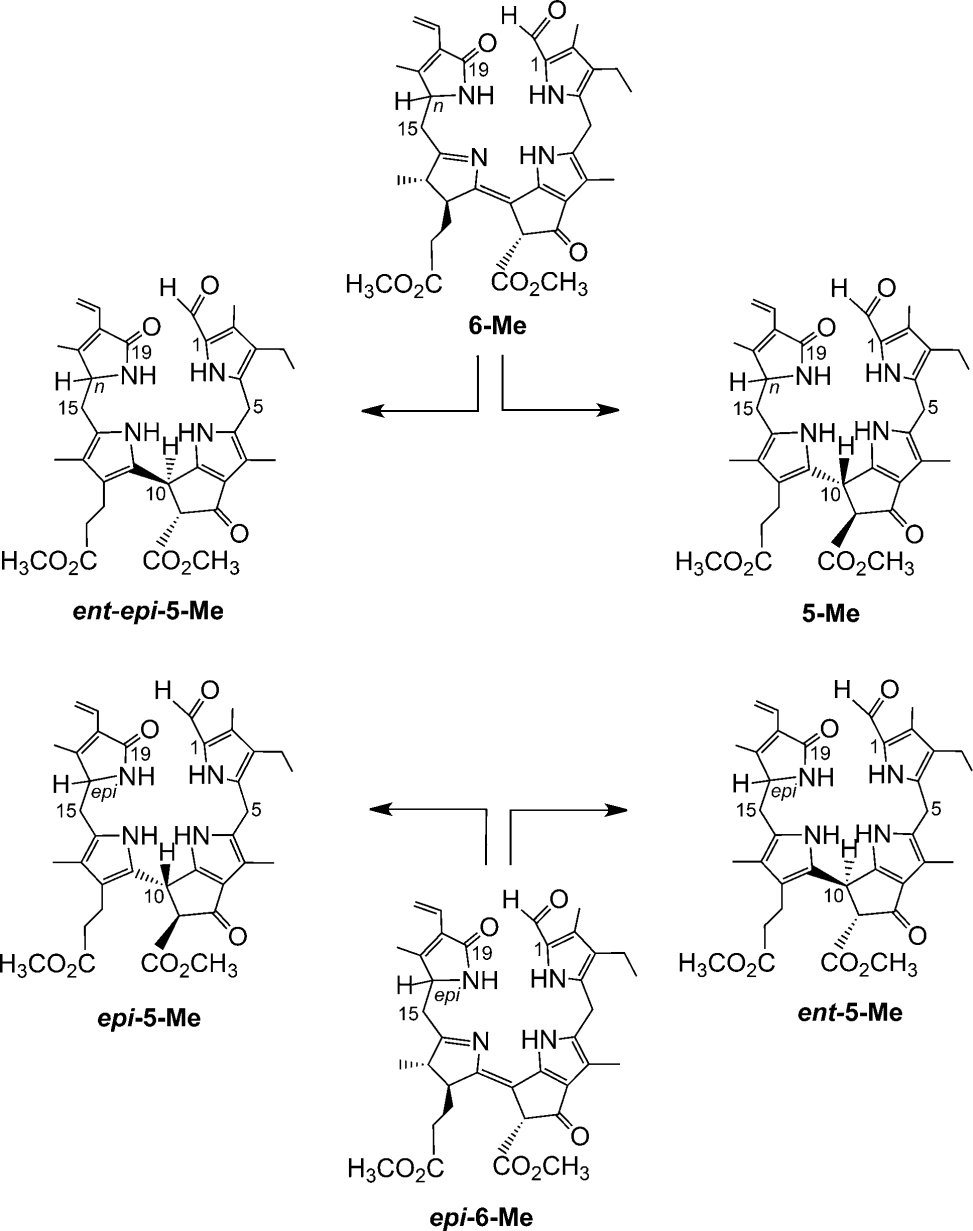
Acid‐induced isomerization of methyl esters of primary FCCs (**6‐Me**/***epi*** **‐6‐Me**) to NCCs is slow and lacks significant stereoselectivity; it furnishes methyl esters of their normal and epi lineages **5‐Me**/***epi*** **‐5‐Me**, as well as of both of their enantiomers (***ent***‐**5‐Me**/***ent***‐***epi***‐**5‐Me**).20b

The critical role of the propionic acid side chain in the FCC to NCC isomerization was further demonstrated by studying this type of isomerization reaction with the related FCC methyl esters **6‐Me** and ***epi*** **‐6‐Me**, which were obtained from partial synthesis.20b Both epimeric *p*FCC esters eventually isomerized to the corresponding NCC methyl esters, but with low stereoselectivity at C10, slow reaction rate, and low conversion (**5‐Me**/***ent*** **‐*epi*‐5‐Me** from **6** and ***epi*** **‐5‐Me**/***ent*** **‐5‐Me** from ***epi*** **‐6‐Me** Figure 14).20b Thus, the mirror images of both natural type NCCs are easily accessible (as methyl esters). Clearly, activation of the isomerization by the free propionic acid function is blocked by its esterification. This remarkable finding has helped to rationalize the surprising accumulation of persistent FCCs in ripening bananas (see Section 6), which are FCCs, biologically “caged” with a propionate ester function.28a

So far, natural NCCs were deduced to feature a common *R* configuration at C10. However, two NCCs, recently isolated from leaves of birch trees displayed CD spectra that were essentially mirror images of the CD spectrum of **1**, thus suggesting a reversed configuration at C10.^73^ This finding suggests, first of all, the relevance of alternative ways that may generate NCCs with the reversed configuration at C10. Possibly, the FCC to NCC isomerization is achieved by an enzyme‐controlled, stereochemically different process in birch leaves. The observation of the aberrant configuration in birch NCCs emphasizes the importance of CD spectra for the characterization of new NCCs.

Various colorless fluorescent Chl catabolites (FCCs), or type‐I phyllolumobilins, were detected in extracts of senescent leaves, where they are easily traced by their (blue) fluorescence. As described above, the primary fluorescent Chl catabolites are formed in higher plants by direct enzyme‐catalyzed reduction of protein‐bound RCC (**7**). The absolute configuration at the C16‐position of the epimeric primary FCCs **6** 10, 25 and ***epi*** **‐6** could, so far, not be specified.21a, 47 Therefore, FCCs are classified as belonging either to the normal series (for **6** and *m*FCCs derived from **6**) or to the epi series (***epi*** **‐6** or *m*FCCs derived from ***epi*** **‐6**). The configuration at C16, once introduced at the *p*FCC level, appears to be retained under physiological conditions in *m*FCCs and in their descendants (NCCs, DNCCs, etc.). Therefore, NCCs, DNCCs, etc. also belong either to the normal or to the epi series (Tables 1 and 2 in this section and Table 3 in Section 4.2).

In contrast to the often accumulating NCCs, and with the exception of the unusually persistent hypermodified FCCs (see Section 6),28a typical FCCs exist only fleetingly in leaves of senescent plants, and their structures have only occasionally been assigned.^10^ In addition, primary FCCs appear to be rapidly functionalized further, and the hydroxylation of *p*FCC/*epi*‐*p*FCC at the C3^2^‐position is an early event in Chl breakdown in higher plants, probably taking place in the chloroplasts.9c Consistent with such an early and efficient hydroxylation at the C3^2^‐position in senescent leaves, 3^2^‐hydroxy‐1‐formyl‐19‐oxophyllobilanes (NCC **11** or its C16 epimer, ***epi*** **‐11**) are a typical major NCC fraction in extracts of senescent leaves, but the corresponding analogues lacking the 3^2^‐OH group were not (**5**) or were only rarely (***epi*** **‐5**) detected.20a However, naturally formed *p*FCC (**6**) was detected (as *At*‐FCC‐3) in extracts of senescent *A. thaliana* leaves.^60^ 3^2^‐Hydroxy‐*p*FCC (**23**, 3^2^‐hydroxy‐12,13,16,19‐tetrahydro‐1‐formyl‐19‐oxophyllobilene‐*b*) was likewise found in senescent leaves of the methyl esterase mutant (MES16) of *A. thaliana*, in which activity for the hydrolysis of the methyl ester function of phyllobilins is lacking.^67^ Its epimer, 3^2^‐hydroxy‐e*pi*‐*p*FCC (**e*pi*‐23**, 3^2^‐hydroxy‐12,13,16,19‐tetrahydro‐1‐formyl‐19‐oxo‐e*pi*‐phyllobilene‐*b*), was identified as *Mc*‐FCC‐62 in the peels of ripening bananas.^51^ Two additional polar FCCs were tentatively identified in senescent *A. thaliana* leaves: 3^2^‐hydroxy‐O8^4^‐desmethyl‐12,13,16,19‐tetrahydro‐1‐formyl‐19‐oxophyllobilene‐*b* (*At*‐FCC‐1, **24**) and O8^4^‐desmethyl‐12,13,16,19‐tetrahydro‐1‐formyl‐19‐oxophyllobilene‐*b* (*At*‐FCC‐2, **22**), which are formed by hydrolysis^67^ of the methyl ester function of 3^2^‐hydroxy‐*p*FCC (**23**) and of *p*FCC (**6**), respectively (Table 2).^60^

**Table 2:** Structures of natural fluorescent type‐I phyllobilins (FCCs, top section) and type‐II phyllobilins (DFCCs, *iso*‐DFCC, bottom section): labels R^1,^ R^2^, and R^4^ refer to the generalized formula of FCCs (with atom numbering), shown in Figure 15.

No.^[a]^	R^1^	R^2^	R^4^	C16^[b]^	Provisional names^[c]^	Ref.
**22**	H	H	H	n	*At‐*FCC‐2	60
**6**	H	CH_3_	H	n	*Bn‐*FCC‐2 (*p*FCC)	25, 60
***epi*** **‐6**	H	CH_3_	H	epi	*Ca‐*FCC‐2 (*epi‐p*FCC)	47
**24**	OH	H	H	n	*At‐*FCC‐1	60
**23**	OH	CH_3_	H	n	3^2^‐OH‐*p*FCC	27
***epi*** **‐23**	OH	CH_3_	H	epi	*Mc*‐FCC‐62	51
**23‐Me**	OH	CH_3_	CH_3_	epi	*Mc*‐FCC‐71^[e]^	51
***epi*** **‐41**	OH	CH_3_	5′‐daucyl^[f]^	epi	*Mc*‐FCC‐56	28a
***epi*** **‐42**	OH	CH_3_	4′‐daucyl^[g]^	epi	*Mc*‐FCC‐53	28a, 85
**25**	OH	CH_3_	^[h]^	n	*Sw‐*FCC*‐*62	74
***epi*** **‐25**	OH	CH_3_	^[h]^	epi	*Ma‐*FCC*‐*69	76
***epi*** **‐26**	OH	CH_3_	^[i]^	epi	*Ma‐*FCC*‐*61	28b
***epi*** **‐27**	OH	CH_3_	^[j]^	epi	*Ma‐*FCC*‐*63	76
***epi*** **‐28**	OH	CH_3_	^[k]^	epi	*Ma‐*FCC*‐*64	76
***epi*** **‐43**	O‐Glc^[d]^	CH_3_	5′‐daucyl^[f]^	epi	*Mc*‐FCC‐49	85
***epi*** **‐44**	O‐Glc^[d]^	CH_3_	4′‐daucyl^[g]^	epi	*Mc*‐FCC‐46	85
**45**	O‐Glc^[d]^	CH_3_	H	n	*At_MES_‐*FCC	67
**34**	OH	H	H	n	*At‐*DFCC*‐33*	26c
**35**	OH	CH_3_	H	n		27
**40**	H	CH_3_	H	n	*At_MES_‐*2HM*‐iso‐*DFCC	26a

[a] Compound numbering (see text), R^3^=vinyl. [b] Relative configuration at C16: n=normal, that is, derived from *p*FCC, or epi=epimeric, that is, derived from *epi*‐*p*FCC. [c] *Bn*‐FCC‐2 (from oilseed rape, *B*. *napus*),^25^ *Ca*‐FCC‐2 (from sweet pepper, *C. annuum*),^47^ *At*‐FCCs (from *A. thaliana*),^60^ *Mc*‐FCCs (from banana peels),28a, 51 *Ma*‐FCCs (from banana leaves, *M*. *acuminata*, Cavendish cultivar),28b, 76 and *Sw*‐FCC‐62 (from senescent leaves of *S. wallisii*).^74^ [d] Glc=β‐glucopyranosyl. [e] Apparently an artefact from the methanolysis of persistent FCC daucyl esters. [f] Daucic acid bound at 5′‐OH. [g] Daucic acid bound at 4′‐OH. [h] R^4^=6‐β‐glucopyranosyl‐(1‐1′)‐2‐[3,4‐dihydroxyphenyl]ethyl. [i] R^4^=6‐α‐galactopyranosyl‐(1‐6)‐β‐galactopyranosyl‐(1‐1)‐glyceryl. [j] R^4^=6‐β‐glucopyranosyl. [k] R^4^=6‐α‐glucopyranosyl.

**Figure 15:**
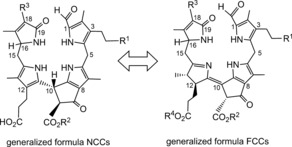
Structures of hypothetical modified FCCs (*m*FCCs, right) are frequently extrapolated from those of the corresponding isomeric NCCs (left).

The actual in vivo presence of (other) modified FCCs with a free propionic acid function has rarely been documented. Therefore, the intermediate natural existence of other *m*FCCs has, so far, been deduced from structure determination of the various natural NCCs, the presumed direct isomerization products of correspondingly modified *m*FCCs (Figure 15).6b, 9c, 10 The NCC structures suggest a range of corresponding FCC modifications that are probably (or are known to be) introduced by cytosolic enzymes.9c Interestingly, the unique modification of *At*‐NCC‐3 (**15**) at C2 indicated a divergent catabolic process in *A. thaliana*,^66^ which was rationalized by a side chain hydroxylation of *p*FCC (**6**) lacking exclusive selectivity for C3^2^.

**Figure 16:**
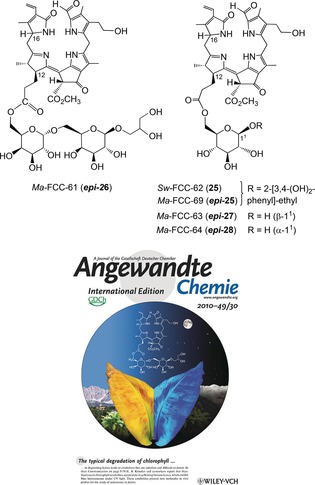
Top: Structural formulas of *hm*FCCs from leaves of bananas (*Ma*‐FCCs) and of *Sp. wallisii* (*Sw*‐FCC‐62), and reproduction (bottom) of a cover picture depicting a yellow banana leaf when observed under daylight or under black light.28b

In contrast to the fleeting existence of typical FCCs, a strikingly persistent and remarkably abundant FCC (>13 % of the Chl in a green leaf) was found in naturally degreened leaves of the tropical evergreen *Spathiphyllum wallisii*.^74^ This hypermodified FCC (*hm*FCC), named *Sw*‐FCC‐62 (**25**), was esterified with a β‐glucopyranosyl‐(1→1)‐2‐(3,4‐dihydroxyphenyl)ethyl group at the crucial propionyl side chain extending from C12 (Figure 16). As propionate ester functions stabilize *hm*FCCs against their acid‐induced isomerization to NCCs, *hm*FCCs are a remarkable group of biologically “caged” versions of ordinary FCCs.

Persistent *hm*FCCs were actually discovered in banana fruit.28a However, a range of persistent *hm*FCCs also accumulated in senescent (yellow) leaves of banana plants (*Musa acuminata*, Cavendish cultivar), whereas nonfluorescent Chl catabolites (NCCs) were not found. The accumulation of *hm*FCCs in the yellow banana leaves induced a diffuse blue luminescence when the leaves were irradiated with UV light at *λ*≈360 nm. A first polar *hm*FCC from the banana leaves (*Ma*‐FCC‐61, ***epi*** **‐26**) was characterized as the propionyl ester of a 6‐α‐galactopyranosyl‐(1→6)‐β‐galactopyranosyl‐(1→1)‐glyceryl moiety (Figure 16).28b This remarkable ester functionality represents the polar head group of membrane components abundant in the thylakoids. *Ma*‐FCC‐61 (***epi*** **‐26**) was, thus, suspected to be an adventitious cleavage product of so far unidentified, more complex, FCC‐type pigments.28b Indeed, the α‐galactopyranosyl‐(1→6)‐β‐galactopyranosyl‐(1→1)‐glyceryl unit is bound strongly by lipases that hydrolyze the ester functions of digalactosyldiacylglycerols with loss of the polar head group.^75^ A small additional group of less‐polar *hm*FCCs from the banana leaves was identified subsequently and turned out to represent FCC esters with β‐glucopyranosyl units attached at the critical propionate at O6′ (their primary OH group). Among these *hm*FCCs, *Ma*‐FCC‐69 featured a 3,4‐dihydroxyphenylethyl aglycon at its glucopyranosyl ester moiety, thus representing the C16 epimer (***epi*** **‐25**)^76^ of *Sw*‐FCC‐62 (**25**) from leaves of *Sp. wallisii*.^74^ This normal/epi stereodivergence is due to the differing classes of RCC reductases: in banana leaves (and, likewise, in banana fruit; see Section 6), a reductase of the RCCR‐2 type is present, whereas in *Sp. wallisii*, an RCCR‐1 produces colorless Chl catabolites of the normal series.^74^

FCCs accumulate in a striking abundance in senescent banana leaves, but NCCs were not detected, nor was there any indication of the presence of type‐II phyllobilins.^76^ In the two less‐polar *hm*FCCs from banana leaves, *Ma*‐FCC‐63 (***epi*** **‐27**) and *Ma*‐FCC‐64 (***epi*** **‐28**), a β‐ or an α‐glucopyranosyl moiety, respectively, was attached through the primary 6′‐OH group. The free anomeric center of ***epi*** **‐27** and ***epi*** **‐28** allowed for the mutual interconversion by spontaneous anomerization in aqueous solution.^76^ The presence of the latter anomeric *hm*FCCs, ***epi*** **‐27** and ***epi*** **‐28**, in extracts of senescent banana leaves strengthened the suggestion that the observed *hm*FCCs could either be precursors, or, possibly, remnants or partial degradation products of still elusive further functionalized *hm*FCCs. Esterification of the critical propionate function of FCCs by glucopyranosyl or, alternatively, by galactopyranosyl groups provides two distinct lines among the persistent banana leaf *Ma*‐FCCs. The sugar units of these *hm*FCCs provide attachment sites for further groups. Indeed, several minor, still less polar FCC fractions were recently analyzed structurally, and were revealed to be *hm*FCCs, formally derived from ***epi*** **‐27**/***epi*** **‐28**, but functionalized further by unusual terpenoid aglycons with β‐glycosidic linkages.^77^

### 4.2 The Type‐II Phyllobilin Branch—Colorless 1,19‐Dioxobilins

**Figure 17:**
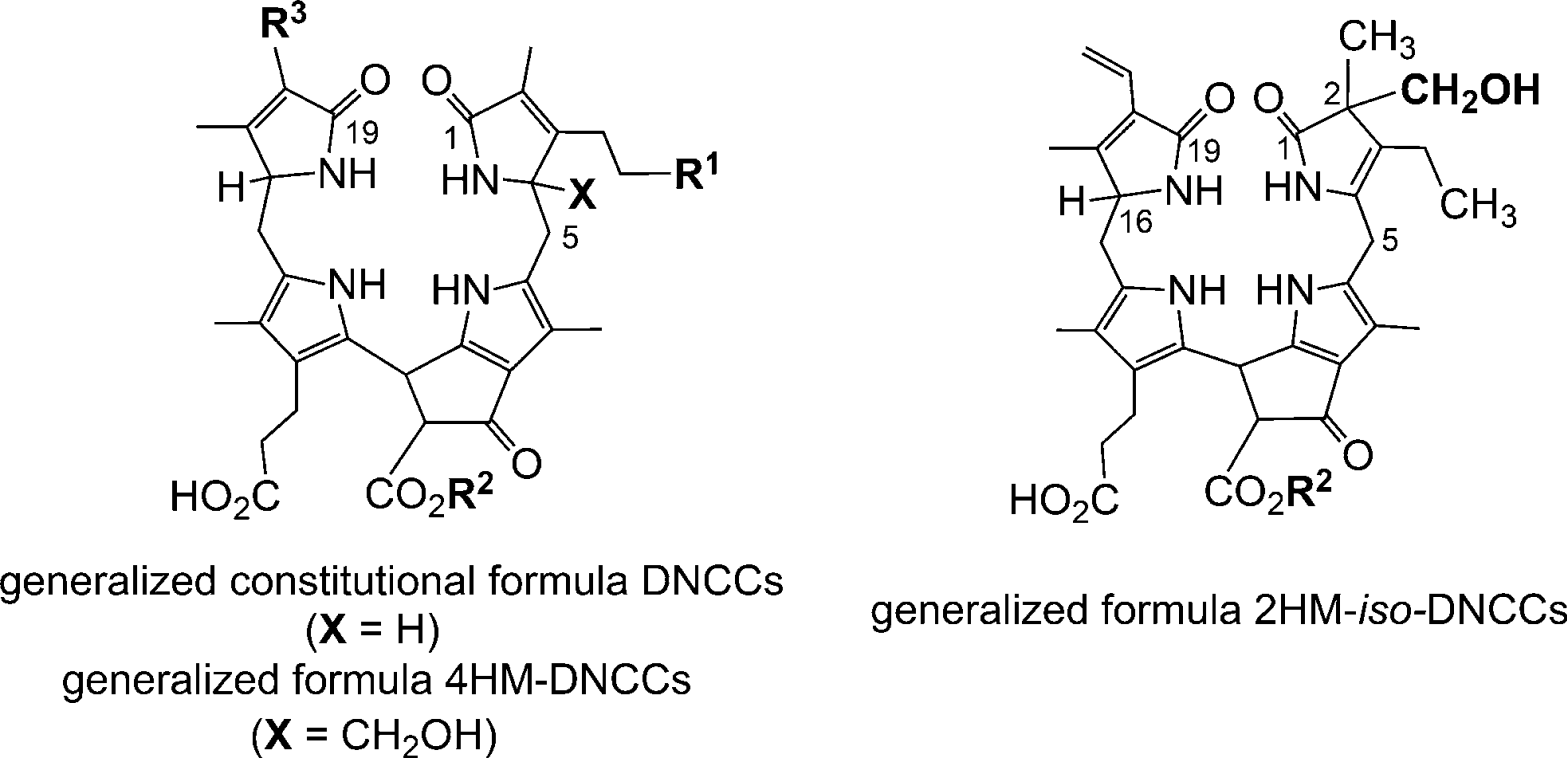
Generalized formulas of colorless and nonfluorescent type‐II phyllobilins: DNCCs, 4‐hydroxymethyl‐DNCCs and 2‐hydroxymethyl‐*iso*‐DNCCs.^26^

The discovery of 1,19‐dioxobilin‐type NCCs (DNCCs) raised the question of their formation and of their natural relevance in Chl breakdown.^57^, ^58^ As products of the PaO/phyllobilin pathway, the native phyllobilins are 1‐formyl‐19‐oxobilins (or type‐I phyllobilins).9c, 10 DNCCs, the 1,19‐dioxobilin‐type Chl catabolites, are the offspring of a subsequent step of Chl breakdown and are, thus, classified as type‐II phyllobilins. Interestingly, the 1,19‐dioxobilin‐type structure of DNCCs (Figures 11 and 17)^57^, ^58^ makes them look remarkably similar to the heme‐derived bilins, the hemobilins.^3^ Recent studies have revealed a striking abundance and constitutional and stereochemical variety of DNCCs that rivals that of the now better studied NCCs, and populating a growing second branch of phyllobilins, of the type‐II phyllobilins.^10^

1,19‐Dioxobilin‐type nonfluorescent Chl catabolites (DNCCs) are formal products from an oxidative removal of the formyl group of NCCs.^57^ The lack of the characteristic absorption of NCCs at *λ*≈315 nm (Figure 9) makes DNCCs more difficult to detect by their UV absorption. Such colorless dioxobilin‐type Chl catabolites (DCCs) accumulate in a variety of senescent leaves. DCCs may occur in leaves together with type‐I phyllobilanes, as is the case in senescent leaves of *A. thaliana*. In wild‐type *A. thaliana* leaves, the polar 1,19‐dioxophyllobilane *At*‐DNCC‐33 (**30**) is, by far, the most abundant phyllobilin, with isomeric *At*‐DNCC‐45 (**31 a**) and *At*‐DNCC‐48 (**31 b**) being minor components.^27^ The 1,19‐dioxophyllobilanes *At_MES_*‐DNCC‐47 (**32**) and *At_MES_*‐DNCC‐38 (**33**) were found in extracts of the *A. thaliana* MES16 mutant (besides minor NCC fractions; Table 3).26a Bilane **33** is a C16 epimer of *Vv*‐DNCC‐51 (***epi*** **‐33**) from degreened grape wine leaves.^78^ The isomeric DNCCs **8 a**, **8 b**, and ***ent*** **‐8 a** (Figure 10 and Table 3) were discovered in senescent leaves of barley^57^ and of Norway maple.^58^

**Table 3:** Structures of natural dioxobilane‐type nonfluorescent Chl catabolites (DNCCs, top section) and of *iso*‐DNCCs (bottom section): labels R^1^, R^2^, and R^3^ refer to the general constitutional formula of DNCCs and *iso*‐DNCCs, shown in Figure 17.

No.^[a]^	R^1^	R^2^	R^3^	C16^[b]^	Provisional names^[c]^	Ref.
**31 a/31 b**	H	H	CH=CH_2_	n	*At*‐DNCC‐45/*At*‐DNCC‐48	26c
**32**	H	CH_3_	CH=CH_2_	n	*At_MES_‐*DNCC‐47	26a
**33**	OH	CH_3_	CH=CH_2_	n	*At_MES_‐*DNCC‐38	26a
***epi*** **‐33**	OH	CH_3_	CH=CH_2_	epi	UNCC‐*Pvir/Vv‐*DNCC*‐*51	78
**30**	OH	H	CH=CH_2_	n	*At‐*DNCC‐33/*Bo*‐DNCC‐3	27, 61
**8 a**	OH	CH_3_	CH(OH)‐CH_2_OH	n	–	57
**8 b**	OH	CH_3_	CH(OH)‐CH_2_OH	n	–	57
***ent*** **‐8 a**	OH	CH_3_	CH(OH)‐CH_2_OH	epi	*Ap*‐DNCC	58
**36**	H	H	CH=CH_2_	n	*At*‐2HM‐*iso*‐DNCC‐43^[d]^	26b
**37**	H	H	CH=CH_2_	n	*At*‐4HM‐DNCC‐41^[e]^	26b
**38**	H	CH_3_	CH=CH_2_	n	*At_MES_‐*2HM‐*iso*‐DNCC‐46^[c]^	26a
**39**	H	CH_3_	CH=CH_2_	n	*At_MES_‐*4HM‐DNCC‐44^[d]^	26a

[a] Compound number (see text). [b] Configuration at C16: n=normal, if derived from *p*FCC, or epi=epimeric, if derived from *epi*‐*p*FCC; configuration at C4 not determined. [c] *Ap*‐DNCC (from *Acer platanoides*);^58^ *At*‐DNCCs (from *A. thaliana*, wild type)26b or *At_MES_*‐DNCCs (from *A. thaliana*, *MES*16 mutant).26a [d] Hydroxymethyl group at C2; UNCC‐*Pvir/Vv‐*DNCC*‐*51(from grape wine).^78^ [e] Hydroxymethyl group at C4 (see Figure 17 for generalized formulas of DNCCs and *iso*‐DNCCs).

The observation of 1,19‐dioxobilin‐type NCCs (DNCCs) first raised the question of their formation and of their general metabolic relevance for natural Chl breakdown.^57^, ^58^ Based on a stereochemical divergence indicated by the deduced structure of the DNCC ***ent*** **‐8 a** from leaves of Norway maple, a split of the PaO/phyllobilin pathway at the level of the fluorescent Chl catabolites (FCCs) was proposed, which, in consequence, would involve the intermediate existence of one (or of several) 1,19‐dioxobilin‐type fluorescent Chl catabolite (or DFCC) intermediate(s).^58^

**Figure 18:**
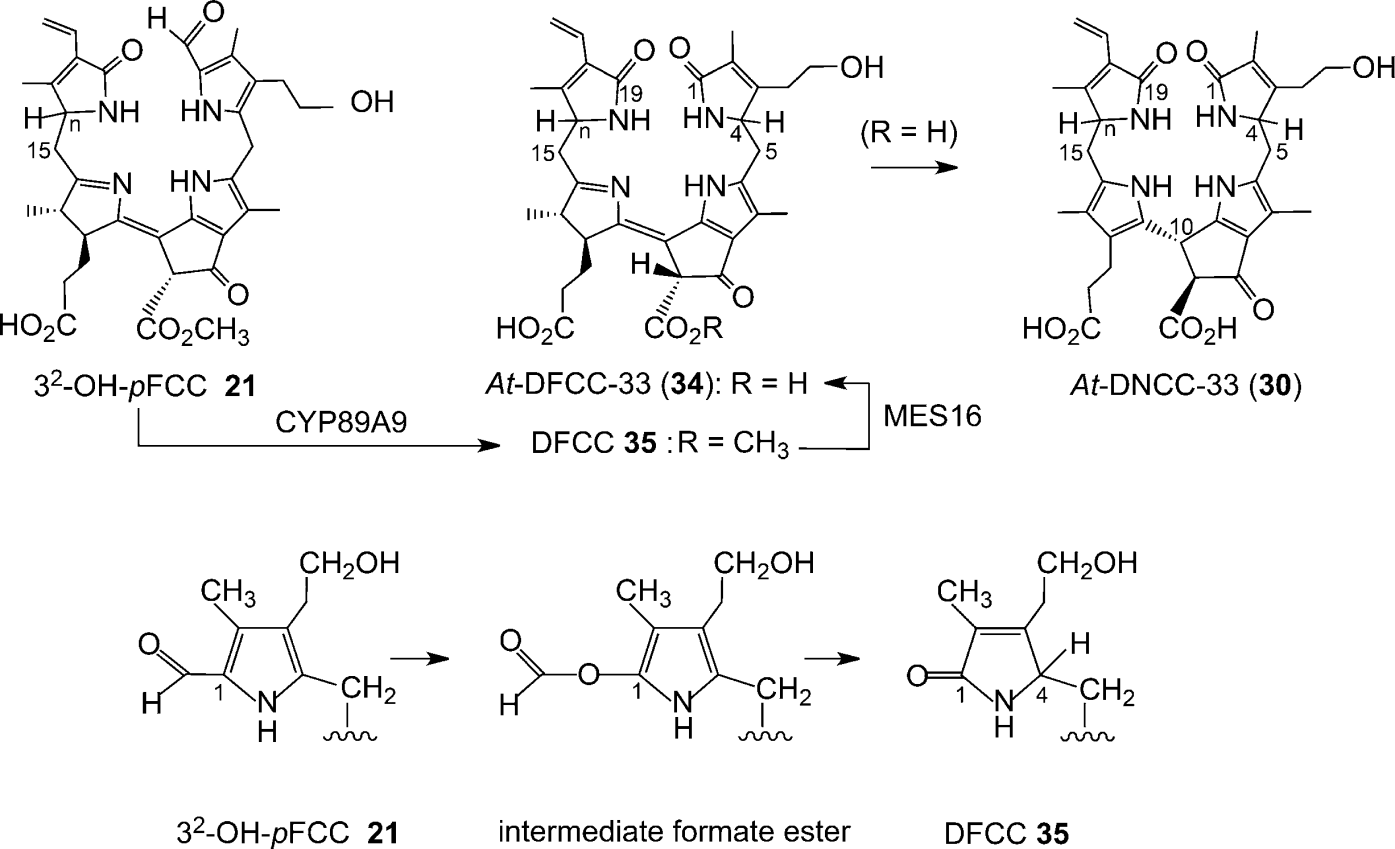
Top: Deformylation of 3^2^‐OH‐*p*FCC by CYP89A9 is proposed as an entry to type‐II phyllobilins by furnishing the hypothetical DFCC **35**; ester hydrolysis by MES16 produces DFCC **34** (*At*‐DFCC‐33), which isomerizes to DNCC **30** (*At*‐DNCC‐33).26c Bottom: Abridged outline of a possible mechanism (depicted by ring A) of the deformylation of FCC **21** to DFCC **35**, catalyzed by the cytochrome P450 enzyme CYP89A9.26a

Indeed, a cytochrome P450 enzyme (CYP89A9) was recently identified in *A. thaliana* that catalyzed the in vitro deformylation of *epi*‐*p*FCC (***epi*** **‐6**), thereby furnishing four epimeric DFCCs.^27^ A pair of these DFCC epimers isomerized rapidly to a pair of DNCCs in weakly acidic solution. These in vitro experiments clarified the basic constitutional features of an FCC deformylation and of the DFCC to DNCC isomerization, proposed to be early key steps of the dioxobilin branch of Chl breakdown.^27^ Clearly, the intriguing deformylation by the cytochrome CYP89A9 requires further investigation. General precedence for the removal of formyl (or acyl) groups by P450 enzymes exists.^79^ However, there appears to be none for a P450‐catalyzed oxidative deformylation at an α‐position of a pyrrole unit. The inferred nucleophilic (hydro)peroxo‐Fe^III^ intermediate of the P450 cycle was suggested to induce oxidative (C−C) bond cleavages.79b, 80 This would thus imply an insertion of oxygen atom into the previous (C−C) bond with formation of a formate ester, reminiscent of the Baeyer–Villiger reaction (Figure 18, bottom). Hydrolysis of this putative ester, removal of the currently unknown C1 fragment (possibly formic acid), and protonation at C4 could all take place without assistance by the P450 enzyme, thereby helping to explain the lack of stereoselectivity observed in the in vitro experiment with CYP89A9.^27^

Thus, the remarkable in vitro results with CYP89A9 did not provide a firm conclusion with respect to the stereochemical outcome of the DFCC/DNCC isomerization, nor was a major step of the natural dioxobilin pathway clearly identified. Careful analysis of an extract of *A. thaliana* leaves at an early degreening stage, revealed a minor (blue) fluorescent fraction exhibiting a characteristic band at *λ*=360 nm (from the conjugated B/C part corresponding to FCCs), but lacking the absorption at *λ*=320 nm of an α‐formylpyrrole unit (Figure 8). A sample of the fleetingly existent natural DFCC **34** (a 3^2^‐hydroxy‐1,4,12,13,16,19‐hexahydro‐1,19‐dioxophyllobilene‐*b*) was recently isolated.26c The structure of **34** was deduced from mass spectrometric and extensive NMR‐spectroscopic analysis.26c The DFCC **34** readily underwent acid‐induced, stereoselective isomerization at pH 5 to generate a single DNCC (Figure 18). The isomerization product was identified as *At*‐DNCC‐33 (**30**),26c the major natural DNCC in senescent leaves of *A. thaliana*.^27^ The DFCC **34** was proposed to be the hydrolysis product of the corresponding, still elusive, methyl ester (DFCC **35**), generated by CYP89A9‐catalyzed deformylation of 3^2^‐OH‐*p*FCC (**23**).26c Hence, a naturally existing, functionalized DFCC was identified and, simultaneously, an important natural branching point to the type‐II phyllobilins was revealed in a higher plant.

## 5 Chl Breakdown in Arabidopsis thaliana—A Model Case

The growing systematic biological knowledge concerning *Arabidopsis thaliana* has also become an important resource in the field of Chl breakdown,^27^ assisting the identification of a number of enzymes in this model plant,9c, 11a in fruitful synergy with our recent complementary work concerned with the discovery and structure elucidation of an extraordinary number of Chl catabolites.^26^, ^27^, ^60^, ^67^ A range of colorless type‐I phyllobilins were found in earlier analyses of extracts of senescent leaves of (wild‐type) *A. thaliana*, including five *At*‐NCCs (**3**, **4**, **15**–**17**,^60^, ^66^ see Table 1) and three *At*‐FCCs (**6**, **22**, **24**, Table 2).^60^ More recently, colorless type‐II phyllobilins were discovered in *A. thaliana*, and the *At*‐DNCCs **30**, **31 a**, and **31 b** were characterized (Table 3),26b, 27 as well as the fleetingly existent DFCC **34**.26c

**Figure 19:**
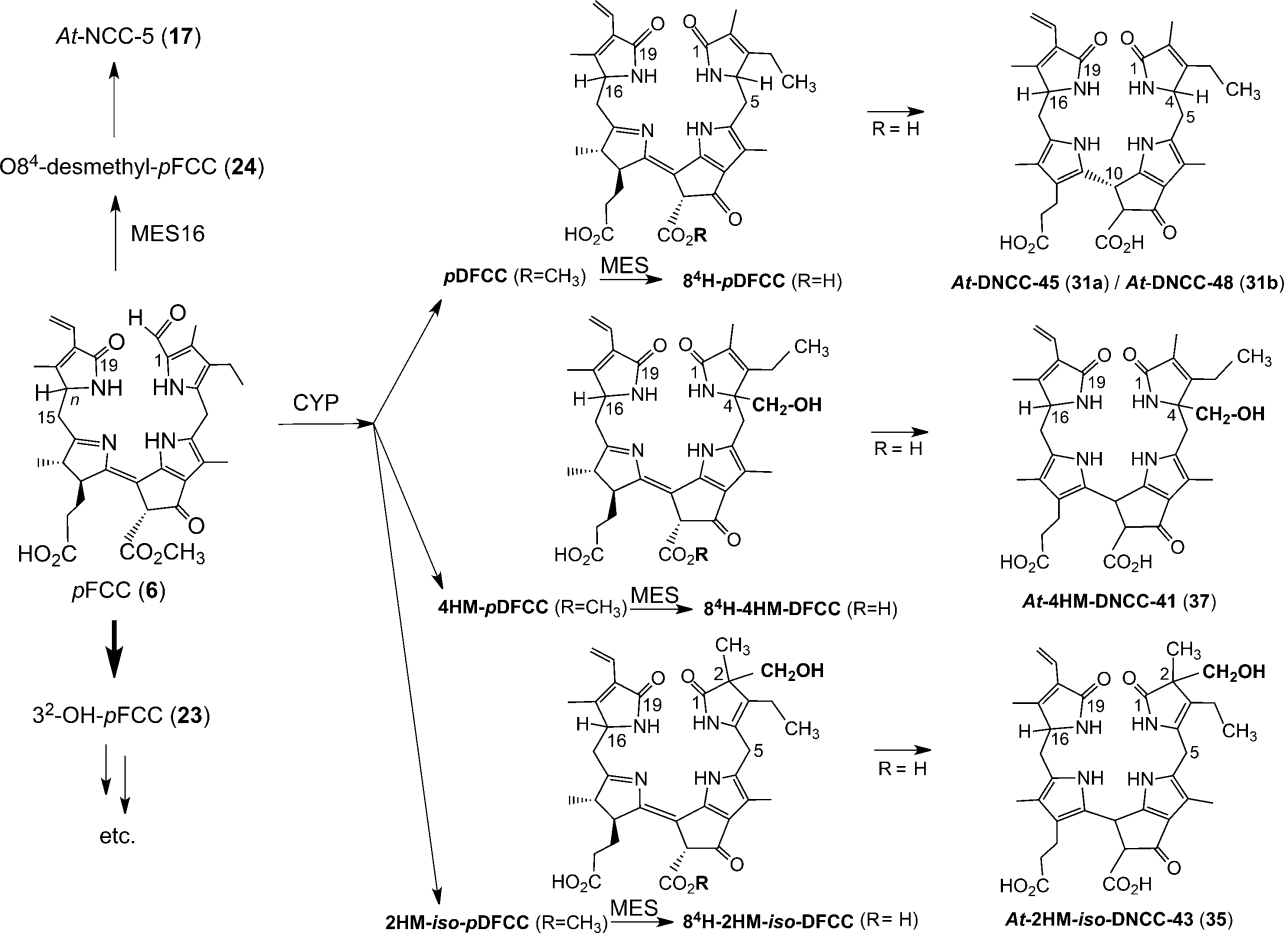
Phyllobilins lacking a 3^2^‐OH group are directly derived from *p*FCC (**6**). In vivo deformylation of *p*FCC(**6**) in *A. thaliana* by CYP89A9 (CYP) and hydrolysis by the methyl esterase MES16 (MES) is a pathway to three types of nonfluorescent DCCs (right: the genuine DNCCs **31 a**/**31 b**, 2HM‐*iso*‐DNCC **35**, 4HM‐DNCC **37**), proposed to be formed by isomerization of the corresponding hypothetical fluorescent DCCs with R=H (center: 8^4^H‐*p*DFCC, 8^4^H‐2HM‐*iso*‐DFCC, and 8^4^H‐4HM‐DFCC).26b

However, various further colorless, nonfluorescent phyllobilins were observed, apparently related to DNCCs, in extracts of senescent leaves of *A. thaliana* (either of wild type26b or of the MES16 mutant26a). These were, provisionally, classified as nonfluorescent DCCs (NDCCs) on the basis of their UV spectra, which were similar to those of the structurally characterized 1,19‐dioxobilin‐type NCCs (DNCCs) **30** and **31 a**/**31 b**. However, as deduced from mass spectra and NMR‐spectroscopic analyses, several of these NDCCs exhibited a puzzling carbon‐hydroxymethylation and, thus, did not have the proper chemical constitution of DNCCs.26a,26b A 2‐hydroxymethyl‐*iso*‐DNCC (*At*‐2HM‐*iso*‐DNCC‐43, **36**) and a 4‐hydroxymethyl‐DNCC (*At*‐4HM‐DNCC‐41, **37**) were discovered (Figure 19 and Table 3)26b in extracts of senescent leaves of wild‐type *A. thaliana*, while the corresponding methyl esters 2‐hydroxymethyl‐*iso*‐DNCC *At_MES_*‐2HM‐*iso*‐DNCC‐46 (**38**) and the 4‐hydroxymethyl‐DNCC *At_MES_*‐4HM‐DNCC‐44 (**39**) were found in extracts of the MES16‐mutant of *A. thaliana*.26a In the leaves of this mutant, an additional minor fraction of a colorless blue fluorescent phyllobilin was noticed, which lacked the *λ*=315 nm band in the UV spectra, but showed the characteristic absorption maximum of FCCs near *λ*=360 nm.26a Thus, this fluorescent compound was tentatively classified as a fluorescent DCC (FDCC). It was isolated and characterized as the 2‐hydroxymethyl‐*iso*‐DFCC *At_MES_*‐2HM‐*iso*‐DFCC (**40**=2HM‐*iso‐p*DFCC, see Figure 19),26a which differed from genuine DFCCs by the constitution of its ring A (Figure 19>). The structural properties of the FDCC **40** suggest its role as the direct precursor of the *At_MES_*‐2HM‐*iso*‐DNCC‐46 (**38**),26a its nonfluorescent isomer. All of these remarkable carbon‐hydroxymethylated type‐II phyllobilins lack an oxygen functionality at their C3^2^‐position, which is typical of most natural phyllobilins.9c, 10 Hence, their structures relate them to *p*FCC (**6**) as their (direct) precursor. Hydroxymethylation appears to be tightly associated with the oxidative deformylation of *p*FCC to type‐II products. Possibly, it is a cytosolic “rescue operation” that introduces a (needed) polar functionality at ring A of the catabolites. In this sense, the hydroxymethylation (at the carbon atom) has been considered a “biosynthetic intermezzo” in the course of the type‐II branch of the PaO/phyllobilin pathway.26a,26b

**Figure 20:**
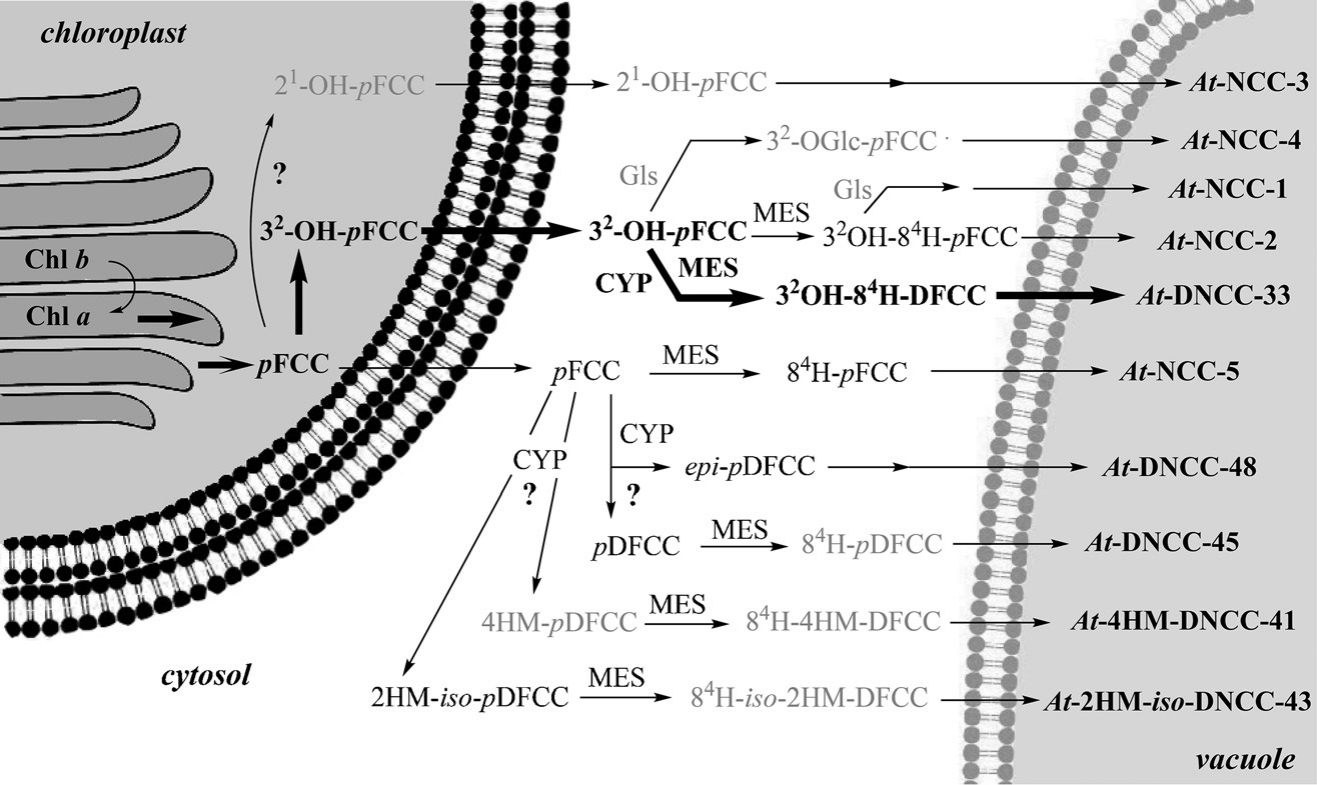
Overview of Chl breakdown in leaves of *A. thaliana* (wild type). The proposed major pathway, from Chl *a* to *At*‐DNCC‐33,26c is marked with bold arrows (CYP=CYP89A9, MES=MES16, Gls=putative glycosidase; see Figure 19 for the structures of type‐II phyllobilins derived from *p*FCC).

In summary, a vast variety of colorless phyllobilins are produced in *A. thaliana* by breakdown of Chl, spearheaded by formation of *p*FCC (**6**) in the chloroplasts, as well as, presumably, of 3^2^‐hydroxy‐*p*FCC (**23**).9c, 26c The descendants of *p*FCC (**6**) that lack an OH group at the C3^2^‐position are minor components among the Chl catabolites in *A. thaliana*, despite their particularly diverse nature (Figures 19 and 20). The major phyllobilins in *A. thaliana* (both the wild type and MES16 mutant) are, instead, derivatives of 3^2^‐hydroxy‐*p*FCC (**23**). Its type‐II phyllobilin descendants dominate over the type‐I analogues. Evidence for export of the FCCs **6** and **23** into the cytosol and for their further independent processing to modified FCCs, DFCCs, and FDCCs is derived from the structures of the FCCs **22**, **24**, DFCC **34**, and FDCC **40**, as well as of their nonfluorescent descendants. The latter are believed to arise by acid‐catalyzed isomerization of their fluorescent precursors after import into the vacuoles. The deduced in vivo deformylations of *p*FCC (**6**) and of 3^2^‐hydroxy‐*p*FCC (**23**) establish two natural branching points from type‐I to type‐II phyllobilins in leaves of *A. thaliana*.26c

## 6 Long Overlooked Chl Catabolites in Fruit and Vegetables

**Figure 21:**
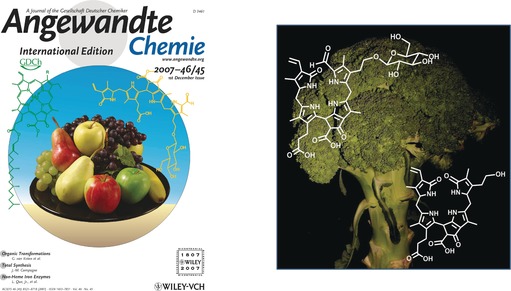
Ripening fruit (left) and degreening florets of broccoli (right) undergo Chl breakdown and accumulate colorless phyllobilins.^59^, ^61^

The disappearance of chlorophyll is commonly associated with the appearance of fall colors. However, ripening fruit (and also vegetables) often undergo degreening processes (Chl breakdown) that are visually similar to those observed in senescent leaves (Figure 21).9c, 11c Hence, the question arose “What happens to Chl when fruit ripen and vegetables degreen?”

### 6.1 Colorless Chl Catabolites in Fruit and Vegetables

As a rule, when apples, pears, or other fruit ripen, the associated appearance of the appetizing colors of the ripe fruit is a visual indicator of their degree of ripeness.^81^ At the same time, the Chl originally present in the unripe green fruits is broken down, presumably to produce phyllobilins. Chl breakdown in the peel of Golden Delicious apples (*Malus domestica*) and of Williams pears (*Pyrus communis*) was shown to yield the nonfluorescent type‐I phyllobilins ***epi*** **‐9** and ***epi*** **‐11**, also named *Md*‐NCCs and *Pc*‐NCCs.^59^ The same (epi‐type, that is, RCCR‐2 derived) NCCs were also found in senescent leaves of the corresponding apple and pear trees, thereby indicating a common pathway in the leaves and fruit of these fruit trees.^59^ Several NCCs, including *Ej*‐NCC‐2 (***epi*** **‐29**, Table 1), were identified (on the basis of mass‐spectrometric and UV‐spectroscopic data) in quince (*Cydonia oblonga*, Miller)^82^ and in loquat fruit (*Eriobotrya japonica*).^83^ Likewise, the NCCs *Bo*‐NCC‐1 (**3**) and *Bo*‐NCC‐2 (**17**), as well as *Bo*‐DNCC‐3 (**30**), were characterized in degreening broccoli florets (*Brassica oleracea*, var. Ital.). These three NCCs are known representatives of the normal stereochemical series of type‐I and type‐II phyllobilins.^61^ Five NCCs were described earlier in senescent spinach leaves (*Spinacia oleracea*), the so‐called *So*‐NCCs (***epi*** **‐1**, ***epi*** **‐5**, ***epi*** **‐11**, ***epi*** **‐12**, ***epi***‐**13**), which belong to the epi series of NCCs.^50^, ^64^ As expected, in ripe(ning) fruit and (degreening) vegetables, Chl breakdown follows the common PaO/phyllobilin pathway and furnishes colorless type‐I and type‐II phyllobilins, in a species‐dependent way. Clearly, these plant‐derived components of our food are a common source of Chl catabolites, which, hence, are part of our daily nutrition.6a

### 6.2 Persistent Blue Luminescent Chl Catabolites in Bananas

**Figure 22:**
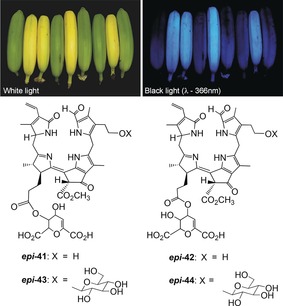
Top: Yellow ripe bananas show a blue luminescence. When yellow bananas are illuminated with UV light (black light), a blue glow of the bananas originates from the abundant FCCs and can be seen by the naked eye^86^ (picture taken from Ref. 28a). Bottom: Major *hm*FCCs from banana peels are FCC daucyl esters.28a, 85

The ripening of bananas is associated with the typical development of a bright yellow color, which, in turn, is commonly considered a critical visual indicator of the degree of ripeness of the banana fruit. Clearly, during the degreening process, the Chl present in the peels of the unripe fruit is degraded. We were, therefore, intrigued to analyze bananas for Chl catabolites.28a Surprisingly, some of the catabolites found in the peels of freshly ripe bananas (*Musa acuminata*, Cavendish cultivar) were revealed to be persistent FCCs and to belong to the then unprecedented group of hypermodified FCCs (*hm*FCCs). These FCCs make the ripening bananas glow blue, as is best seen when analyzed by irradiation with black light and observation in a dark room (Figure 22).28a Several persistent *hm*FCCs accumulate in the peels of ripe(ning) bananas, where the *Mc*‐FCCs ***epi*** **‐23**, ***epi*** **‐41**, and ***epi*** **‐42** represent a sizeable fraction of the phyllobilins. The major *hm*FCCs of the peel of ripe bananas, *Mc*‐FCC‐56 (***epi*** **‐41**) and *Mc*‐FCC‐53 (***epi*** **‐42**), feature an ester at the propionic acid function that is derived from daucic acid^[84]^ itself.28a The more polar *Mc*‐FCC‐49 (***epi*** **‐43**) and *Mc*‐FCC‐46 (***epi*** **‐44**) are further modified at their 3^2^‐positions by a β‐glucopyranosyl unit (Table 2 and Figure 22).28a, 85 Thus, *Mc*‐FCCs differ characteristically from related *hm*FCCs so far found in senescent leaves of bananas^51^ or of the Peace Lily,^74^ which are all esterified with typical natural hexopyranosyl units.

**Figure 23:**
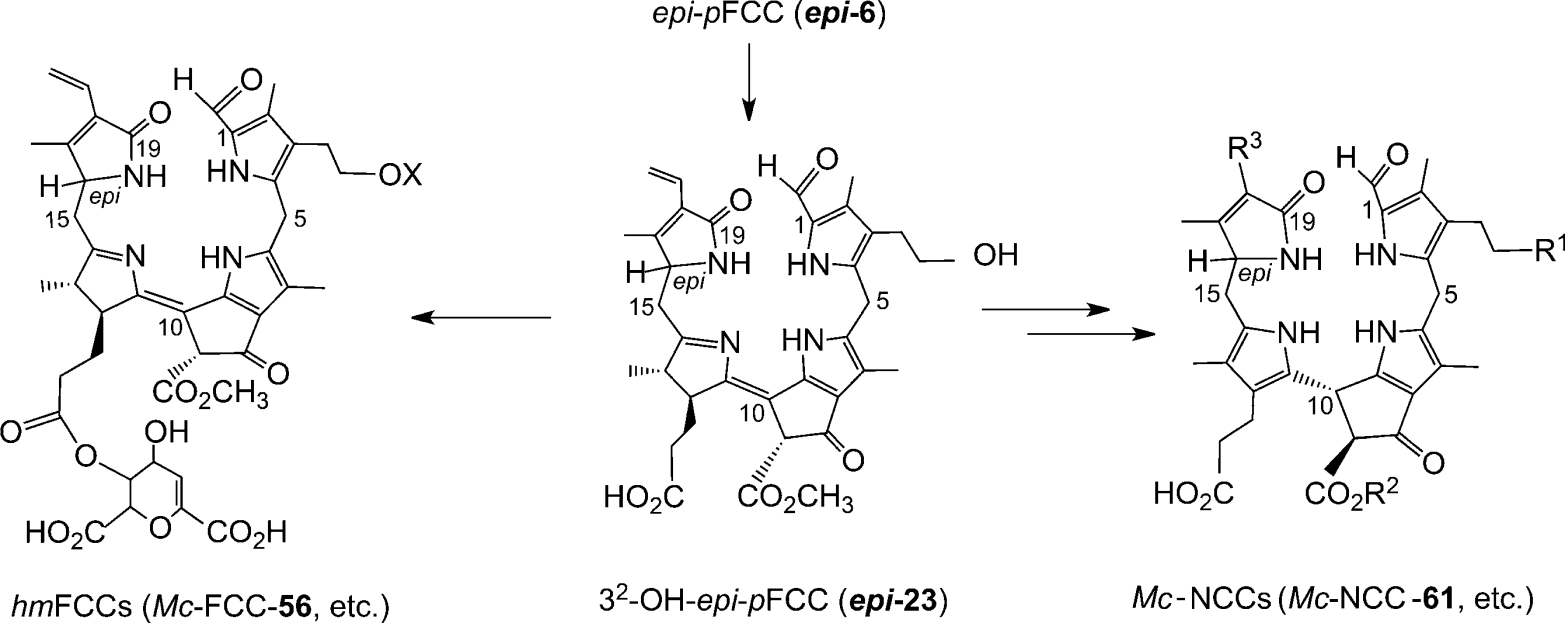
*hm*FCCs and NCCs are generated in the peels of ripening bananas, thereby indicating a pathway of Chl breakdown that is split at the stage of 3^2^‐OH‐*epi‐p*FCC (***epi*** **‐23**).^51^

In the early phase of the ripening process of bananas, the classical FCC 3^2^‐hydroxy‐*epi*‐*p*FCC (*Mc*‐FCC‐62, ***epi*** **‐23**) could also be observed as a major FCC fraction, the rational precursor of the hypermodified FCCs (*hm*FCCs) in the banana peel.^51^ In addition, a variety of *Mc*‐NCCs were characterized in extracts of the banana peels. However, similar to the banana leaves,^76^ representatives of the type‐II phyllobilins were not detected.^51^ These findings indicated an exclusive role of type‐I phyllobilins (of the epi‐type) in banana peels, as well as a pathway of Chl breakdown that diverges into two branches of type‐I phyllobilins at the level of FCCs (Figure 23). This stage of Chl breakdown is presumed to be located in the cytosol, where formation of specific *hm*FCCs through esterification of 3^2^‐hydroxy‐*epi*‐*p*FCC (***epi*** **‐23**) with a daucyl group competes with other modifications that furnish typical *m*FCCs. The latter are presumed to be transported into the vacuoles for eventual rapid isomerization to *Mc*‐NCCs (Table 1).^51^

As is commonly observed, dark, senescence‐associated spots develop naturally on the peels of very ripe bananas.^87^ This deterioration can be inhibited by protection from air (or oxygen).^88^ These spots arise around the stomata as a sign of local senescence and eventual cell death.^85^ Strangely, glucosylated *hm*FCCs (especially ***epi*** **‐43** and ***epi*** **‐44**) accumulate specifically in the senescent area around the dark spots. This local FCC enrichment in areas encircling the growing necrotic spots can easily be observed in darkened rooms, when black light is used as the light source.^85^ The blue fluorescent rings observed on the peels of overripe bananas arise in areas committed to programmed cell death, and have, thus, been considered to represent “blue halos of cell death”.^85^

## 7 Phyllochromobilins from Oxidation of Phylloleucobilins

**Figure 24:**
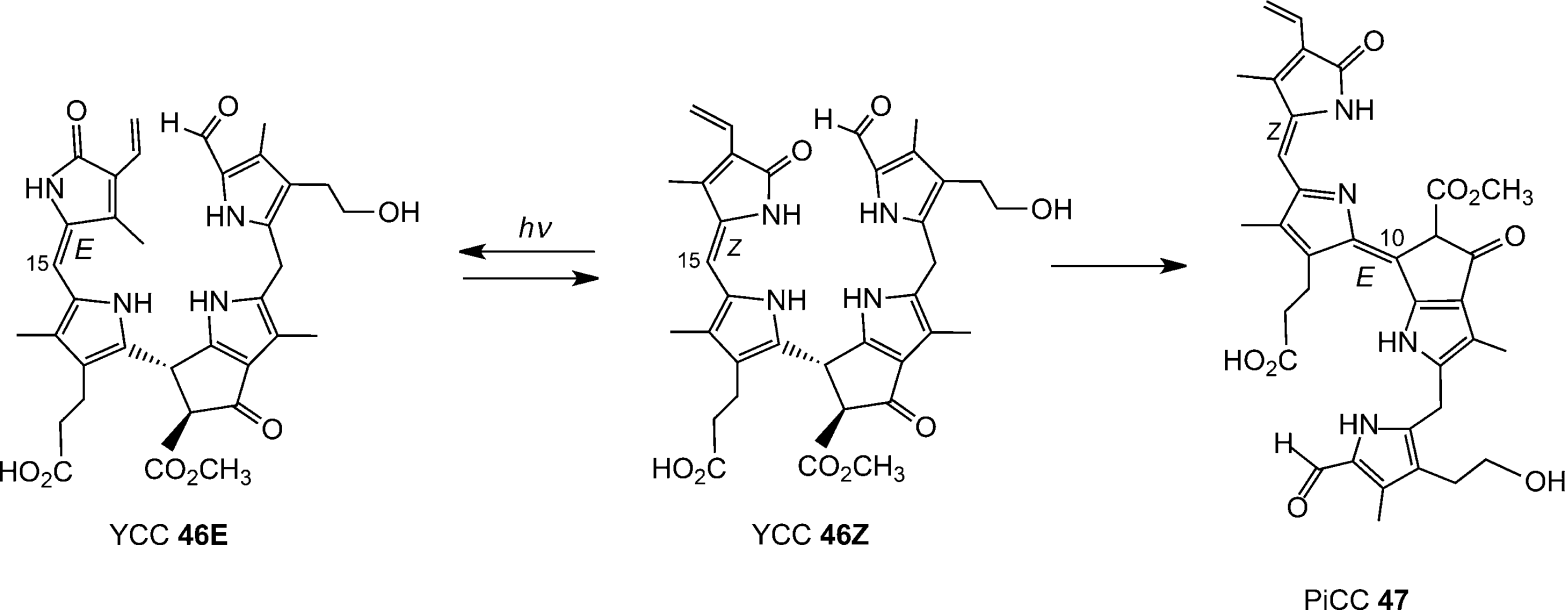
Structural formulas of yellow Chl catabolites (YCCs) and pink Chl catabolites (PiCCs), which may contribute to the color of senescent leaves of deciduous trees.^89^

Early on, NCCs were called “rusty pigments”.8a, 14 Indeed, samples of these colorless products of Chl catabolism, which often accumulate as apparently final products of Chl breakdown in senescent leaves of higher plants, readily become rust colored.^7^ Analysis of such a colored mixture obtained by exposure of a solution of the common NCC ***epi*** **‐11** to sunlight in the presence of air revealed the presence of the yellow compounds **46Z** and **46E**, as well as of pink‐colored **47**, which we classified as yellow Chl catabolites (YCCs) and pink Chl catabolites (PiCCs), respectively (Figure 24).6b, 7, 89

### 7.1 Yellow Phyllobilins—The Phylloxanthobilins

Intriguingly, the *E*/*Z*‐isomeric YCCs **46Z** and **46E** were identified in freshly prepared extracts of yellow *Cercidiphyllum japonicum* leaves. This observation supports the actual presence of such phyllochromobilins in senescent leaves and indicates their contribution to the color of senescent leaves.^7^ In polar solution, YCCs show a typical absorption band at *λ*≈430–440 nm,^7^ as do bilirubin (BR)^90^ and model dipyrrinones.^3^ When a solution of the YCC **46Z** was exposed to daylight, **46Z** isomerized (in part) to the *E* isomer **46E**.^89^ Structure analysis of YCCs **46Z** and **46E** by heteronuclear NMR spectroscopy indicated an unsaturated “western” *meso* position and, as a consequence, a π‐conjugated chromophore extending over rings C and D. In a formal sense, **46Z** and **46E** are formed from NCC ***epi*** **‐11** by oxidative desaturation with formation of a C15=C16 double bond. This (part of the) chromophore of YCCs features the same remarkable (local) properties as the C/D part of the chromophore of bilirubin (BR).^3^, ^90^ X‐ray analysis of crystals of **46Z‐Me** (the methyl ester of **46Z**) confirmed the structure of **46Z‐Me** (and, thus, of **46Z**) that had been deduced from their spectra.^7^ In addition, it revealed a hydrogen‐bonded dimer of **46Z‐Me** in the crystal and verified the earlier deduced *R* configuration of **46Z** 7 and of its precursor ***epi*** **‐11** 20a at C10.^72^

**Figure 25:**
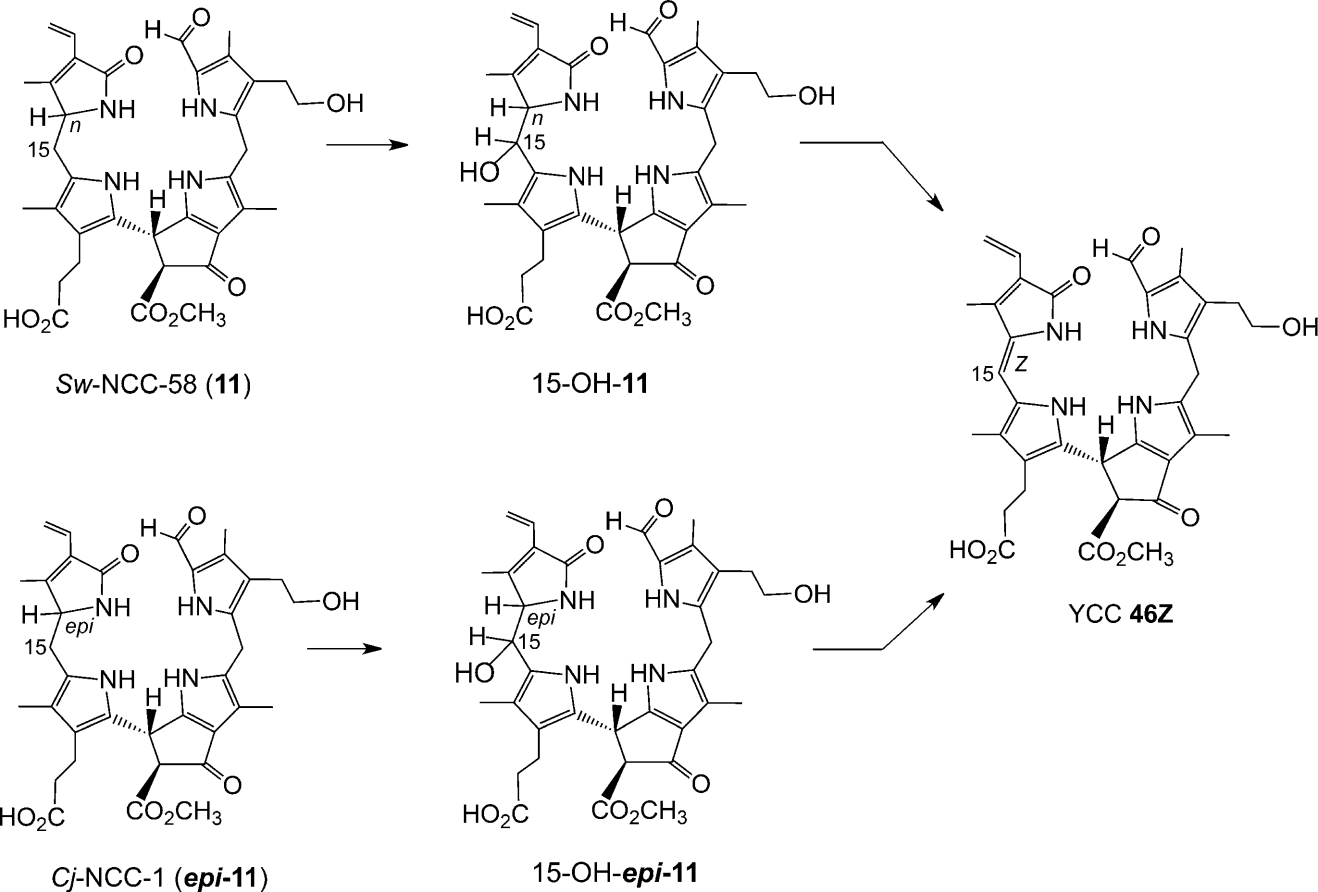
The NCCs **11** and ***epi*** **‐11** are oxidized by an extract from *Sp. wallisii* leaves to epimeric 15‐OH‐NCCs, which are dehydrated in weak acid to furnish the YCC **46Z**.^91^

Oxidation of the NCC ***epi*** **‐11** by dicyanodichlorobenzoquinone (DDQ) opened up a semipreparative pathway to the YCCs **46Z** and **46E**.^89^ In the course of this synthetic transformation, **11** or ***epi*** **‐11** was stereoselectively hydroxylated to 15‐OH‐**11** or to 15‐OH‐***epi*** **‐11**, respectively (Figure 25). Surprisingly, selective oxidation of either endogenous NCC **11** or of added ***epi*** **‐11** also occurred in aqueous homogenates of green or senescent *Sp. wallisii* leaves in the presence of air (or molecular oxygen), with formation of 15‐OH‐**11** or 15‐OH‐***epi*** **‐11**. Both of the oxidized NCCs 15‐OH‐**11** or 15‐OH**‐*epi*‐11** are efficient precursors of the same YCC **46Z**, as a result of selective acid‐induced elimination of water from C15 and C16.^91^ Homogenates of (green or senescent) *Sp. wallisii* leaves contain a still poorly characterized oxidative activity (likely to be enzyme‐based), which provides an entry to the endogenous formation of the YCC **46Z** from **11** or ***epi*** **‐11**, as well as from some other NCCs.^91^ The scope of this type of “green synthesis with leaves” on the basis of their still puzzling oxidative activity remains to be explored, as does the selectivity and preparative limitations of this type of transformation. An analogous oxidation with DNCCs (as observed with NCCs **11** and ***epi*** **‐11** 91), could provide a possible pathway to the corresponding dioxobilin‐type YCCs (DYCCs). YCC‐type compounds were not only detected in senescent leaves of *C. japonicum*,^7^ but also in fresh extracts of a variety of senescent leaves, for example, of the deciduous lime^92^ and *Egeria densa* trees,^93^ as well as in the peel of ripe bananas.^51^

### 7.2 Pink Phyllobilins—The Phylloroseobilins

**Figure 26:**
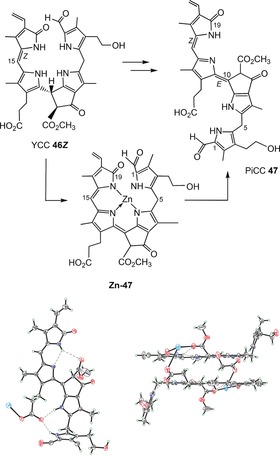
Top: Oxidation of YCC **46Z** in the presence of Zn^II^ ions furnishes the blue Zn^II^ complex **Zn‐47**, from which the PiCC **47** is liberated by treatment with acid or phosphate; bottom: molecular structure of the **PiCC 47** as deduced from X‐ray crystal structure analysis (C gray, green H, red O, blue N; left: top view, right: side view).94a

**Figure 27:**
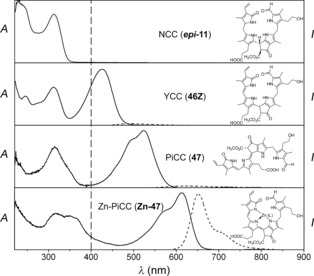
UV/Vis spectra (—, left axis) and fluorescence emission spectra (‐ ‐ ‐ ‐, right axis) of NCC ***epi*** **‐11** (top) and of the phyllochromobilins **46Z** (a YCC) and **47** (a PiCC), as well as of the blue Zn^II^ complex **Zn‐47**.^94^

Similar to bilirubin (BR),^90^ YCCs are easily oxidized in the presence of air or molecular oxygen. Pink Chl catabolites, classified as PiCCs, are obtained from the oxidation of YCCs.^89^ For synthetic purposes, the oxidation of, for example, the YCC **46Z**, can be achieved efficiently in the presence of an excess of Zn^II^ ions, thereby furnishing the bright blue Zn^II^ complex **Zn‐47**.^94^ Treatment of **Zn‐47** with acetic acid or phosphate removes the Zn ion and liberates the PiCC **47** nearly quantitatively (Figure 26).94a The pink phyllobiladiene‐*b*,*c* **47** features a long‐wavelength absorption band at *λ*≈520 nm, consistent with the further extension of the conjugated π system to ring B (Figure 27). The chromophore of the PiCC **47** exhibits a remarkable correspondence to that of the heme‐derived bilin phycoviolobilin.^3^, ^56^ Desaturation of the “southern” C10‐position of the YCC **46Z** caused the PiCC **47** to become available as a racemate: its single asymmetric center (C8^2^) is acidified by adjacent functionalities that assist its fast racemization.94a Detailed NMR‐spectroscopic analysis of the PiCC **47** indicated a striking *E* configuration of the C10=C11 double bond. An X‐ray crystal‐structure analysis confirmed the NMR‐derived structure and revealed the oxidized phyllobiladiene‐*b*,*c* **47** as a pair of hydrogen‐bonded enantiomers in the crystal, which π stack, thanks to their extended planar chromophore system (see Figure 26).^94^

The recent observations of the natural occurrence of YCCs and PiCCs points to a more general relevance of further endogenous transformations of colorless Chl catabolites in senescent plants that go beyond the stage of the abundant type‐I and type‐II phylloleucobilins. Such endogenous processes may represent important further steps of natural Chl breakdown, thereby helping to explain the eventual pronounced decrease in the amount of colorless phyllobilin‐type Chl catabolites in leaves, frequently noted when analyzing leaves undergoing progressive senescence over several days to weeks.

## 8 Phyllobilins Are Natural Products with Remarkable Properties

Phyllobilins, Chl‐derived linear tetrapyrroles,^10^ have structures related to those of the heme‐derived (hemo‐)bilins.^3^, ^90^ Accordingly, phyllobilins are expected to display diverse photo‐, coordination and redox chemistry. However, the chemical properties of phyllobilins have barely been explored to date.^10^, 94b, 95

### 8.1 Phyllobilins as Photoactive Tetrapyrroles

The photochemical properties of the breakdown products of the green photosynthetic pigment Chl have been of prime interest. The observed rapid catabolic transformation of Chl into colorless phyllobilins has been rationalized primarily as the destruction of potentially phototoxic Chl. Hence, the observed rapid formation of the nonfluorescent and colorless type‐I and type‐II phyllobilins (NCCs and DNCCs) is completely in line with such a detoxification aspect of Chl breakdown. Indeed, NCCs and DNCCs, which typically accumulate in senescent leaves, display absorptions limited to the UV region of sunlight (Figure 8), and they, furthermore, lack photoactivity.

Remarkably, photoactive breakdown intermediates do not typically accumulate during the rapid breakdown of Chl on the way to the nonfluorescent phyllobilins. Fluorescent Chl catabolites, such as *p*FCC (**6**) and 3^2^‐hydoxy‐*p*FCC (**23**), as well as their C16 epimers, represent an important intermediate stage in the PaO/phyllobilin pathway.9c The FCC **6** absorbs very little light in the visible region (Figure 9). As their classification suggests, FCCs are effective emitters of blue fluorescence.28a, 95 Thus, ***epi*** **‐23‐Me**, the semisynthetic methyl ester of 3^2^‐hydoxy‐*epi*‐*p*FCC (***epi*** **‐23**) features an emission with a maximum at *λ*=437 nm and a fluorescence quantum yield of 0.21 (lifetime: 1.6 ns in ethanol).^95^ The photoexcited ***epi*** **‐23‐Me** undergoes intersystem crossing into the triplet state (with a quantum yield of 0.6), from which it generates singlet oxygen with nearly 100 % efficiency. Thus, the FCC ***epi*** **‐23‐Me** is a remarkably potent sensitizer of singlet oxygen (^1^O_2_). However, FCCs exist only fleetingly in senescent leaves, except when “caged” as the persistent hypermodified FCCs (*hm*FCCs).28a The deduced, similar photochemical features of *hm*FCCs are noteworthy, as they may play a physiological role in senescent plant tissue and in ripening fruit (see Section 9).

Colorless NCCs are transformed into the yellow YCCs (e.g. **11** into **46Z**) by desaturation at their C15‐position. Similar to their distant analogue BR,^90^ YCCs absorb blue light (absorption maxima at *λ*≈430–440 nm) and exhibit only a weak emission with a maximum at *λ*≈500 nm (Figure 27), which indicates rapid deactivation of their excited states.94b Similar to BR, the YCC **46Z** undergoes light‐induced *E*/*Z* isomerization to **46E**.^89^ Oxidation and desaturation of **46Z** at the C10‐position produces the PiCC **47**, which absorbs at *λ*≈520 nm, and emits weakly (with a maximum at *λ*≈620 nm, Figure 27).94a

### 8.2 Phyllobilins as Antioxidants

As is typical for bilanes and strongly reduced hydroporphinoids,^96^ NCCs are easily oxidized.^89^ In line with this property, they are also remarkable amphiphilic antioxidants,^59^ as evident by their inhibitory effect in the classical autoxidation reaction of linoleic acid.^97^ In such tests, the NCC ***epi*** **‐11** exhibited an only five times lower capacity than bilirubin (BR),^59^ an effective and physiologically important antioxidant.^97^ Analogous investigations of the effect of the corresponding YCC **46Z** on the autoxidation of linoleic acid indicated this YCC to even inhibit about 3–5 times more effectively than BR, not unexpected in view of the similar features of their chromophores.^98^ Experiences on the antioxidant effect of other phyllobilins (such as model FCCs, DNCCs, and DYCCs) would also be of interest. This aspect of the properties of phyllobilins still remains to be studied.

### 8.3 Phyllobilins as Ligands in Transition‐Metal Complexes

**Figure 28:**
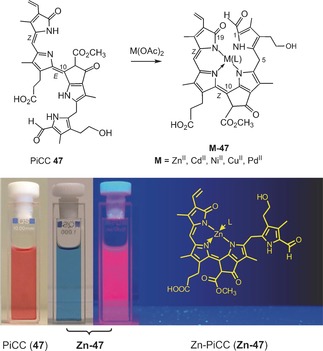
Complexation of transition‐metal ions by PiCC **47** restructures the ligand to a *Z*/*Z* configuration and leads to blue metal complexes. **Zn‐47** and **Cd‐47** are complexes with closed‐shell metal ions, and exhibit strong red luminescence.94a

In contrast to the cyclic tetrapyrroles, which enrich nature with the important metalloporphyrinoid cofactors,^71^, ^99^ a less typical feature of linear tetrapyrroles is their ability to bind metal ions.^3^, ^100^ However, heme‐derived bilins and phyllo(chromo)bilins can be considered to share similar features as multidentate ligands for (transition‐) metal ions.94b, 100 Four nitrogen centers are available in linear tetrapyrroles for the coordination of metal ions.94b However, N atoms of isolated pyrrole rings can hardly compete with polar solvent molecules for coordination at metal ions. Therefore, the photoinactive nonfluorescent NCCs and DNCCs are judged as lacking the capacity for complexing.94b In contrast, the availability of nitrogen atoms of the imine and enamine types in phyllochromobilins provides centers for coordination to transition‐metal ions. So far, the scarcity of such phyllobilins has limited the corresponding studies to the coordination behavior of PiCC **47** and, to some extent, of YCC **46Z**, and its methyl ester **46*Z*‐Me**.^94^ PiCC **47** binds the transition‐metal ions Zn^II^, Cd^II^, Ni^II^, Cu^II^, and Pd^II^ in 1:1 complexes with high affinities and with reaction rates in the order of roughly 100 m ^−1^ s^−1^ (Figure 28). Binding of these transition‐metal ions is easily observed by a strong red‐shift of the absorption bands in the visible region, for example, by Δ*λ*≈100 nm to about *λ*=620 nm for the complex **Zn‐47**. Tridentate binding of the Zn^II^ ion by **47** has been deduced from NMR data in solution and a high affinity (in this case) with a linear 1:1 stoichiometry down to 1 nm solutions of Zn^II^.94a Binding at such low concentrations could be quantified by analysis of fluorescence emission. In contrast to the very weak fluorophore of **47**, the monomeric and diamagnetic complex **Zn‐47** features a strong fluorescence with an emission maximum at *λ*=650 nm (see Figures 27 and 28).94a Coordination by such transition‐metal ions restructures the *E*,*Z*‐phyllobiladiene‐*b*,*c* **47** to a *Z*,*Z*‐structured and effectively tricoordinate ligand (Figure 26).^94^ In **Zn‐47**, the coordinated metal ion also constrains and rigidifies the chromophore part of the ligand, thus inhibiting deactivation pathways through light‐induced *E*/*Z* isomerization, which are presumably available to the free PiCC **47**.94a

**Figure 29:**
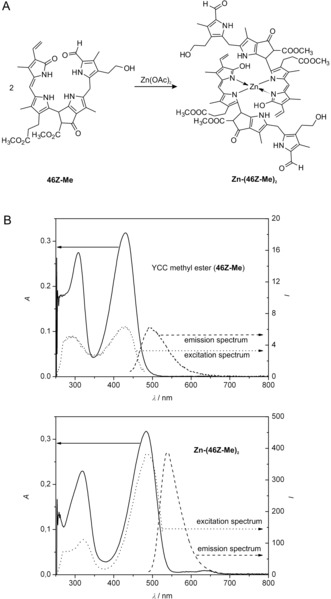
Binding of Zn^II^ ions to the weakly luminescent YCC methyl ester **46Z‐Me** furnishes the strongly fluorescent 2:1 complex **Zn(46Z‐Me)_2_**.94b,94c

The YCC methyl ester **46Z‐Me** effectively behaves as a bidentate ligand for Zn^II^ ions. ^1^H NMR NOE experiments with the diamagnetic Zn complex of **46Z‐Me** suggested interligand distances indicative of a 1:2 arrangement in the symmetrical complex **Zn(46Z‐Me)_2_** (Figure 29).94b,94c Similar to the situation with PiCC **47**, the binding of the closed‐shell Zn^II^ ion to **46Z‐Me** in DMSO solution also shifted the long‐wavelength absorption maximum from *λ*=430 nm to 484 nm. At the same time, the luminescence of **46Z‐Me** (weak emission maximum at *λ*=495 nm) was red‐shifted in the complex **Zn(46Z‐Me)_2_**, with a maximum at *λ*=538 nm, and was also intensified nearly 100‐fold.94b Clearly, the little‐explored behavior of some of the phyllobilins, such as PiCC **47** and YCC **46Z**, in coordinating transition‐metal ions indicates a considerable potential of these bilin‐type ligands in binding metal ions at low concentrations and, thus, in also serving as effective indicators of the presence of metal ions.94a,94b

## 9 On the Role of Chlorophyll Breakdown in Higher Plants—Time for a New Paradigm

Having deciphered, to some extent, how Chl is degraded in some higher plants, we may now be in a better position to address the challenging question of why Chl breakdown occurs.9a,9c Considering the massive amounts of Chl degraded each year on Earth, the phenomenon of Chl breakdown cannot be understood as a mere visual spectacle in nature and as a priceless tourist attraction in some areas of the world.^101^ On the contrary, Chl breakdown should be considered, above all, as having beneficial consequences for the plants themselves.101b Furthermore, other organisms interacting with plants may also benefit (indirectly), such as, for example, animals and humans that make use of plant‐based nutrition. Therefore, and in view of the sheer amount of phyllobilins produced when Chl disappears,^10^ Chl breakdown deserves close scientific attention, not only from fundamental and applied research, but also from basic agrobiological and ecological points of view.^9^, ^10^, ^11^

### 9.1 Chl Detoxification and Labilization of Proteins as Classical Roles of Chl Breakdown

In a historical interpretation, Chl breakdown was given a role in serving the direct recovery of the four nitrogen atoms of the Chl from leaves.^2^ Taking note of the now established build‐up of the phyllobilins as linear tetrapyrroles, this view can, clearly, not be supported any longer.1a, 8a, 9c However, a major direct consequence of Chl breakdown in senescent leaves is the destruction of the abundant and highly photoactive green plant pigment, thus, effectively getting rid of a cellular constituent with a strong potential as a phototoxic agent.9a In addition, removal of the Chls from their proteinaceous binding partners also renders the latter more labile for proteolysis.^102^ This consequence of Chl breakdown obtained strong support from studies with “stay green” mutants (which retain their Chl), in which the relocation of proteinogenic nitrogen atom, and its recovery were found to be strongly reduced. With this knowledge, the ambivalent role of Chl breakdown in plants cannot be overestimated:^2^, ^5^, 101b on the one hand, it eliminates the photosynthetic capacity of Chl, thus reducing a major supply line that drives metabolic activities; on the other hand, by delivering important nutrient components, such as reduced nitrogen, it contributes to enriching protein in crops and vegetables, and to the development of nutritious fruit, both of which are important from an economic point of view.11c, 103

### 9.2 On Physiological Roles of Chlorophyll Catabolites in Plants

Rather than disappearing in senescent leaves “without leaving a trace”,^2^ Chl furnishes the abundant phyllobilins. The latter, instead of being considered mere waste from Chl‐detoxification,1a, 9a should now be given attention with respect to their possible physiological roles in plants. Strikingly, biological functions of bilin‐type Chl catabolites are so far still unknown. This may, in part, be due to a lack of reliable samples of typical Chl catabolites, and to difficulties in their practical application. As a rule, phyllobilins are complex and rather unstable compounds. In this latter respect, the “persistent” *hm*FCCs28a appear to represent a remarkable exception: in *hm*FCCs, the inherent tendency of FCCs to undergo isomerization to the corresponding NCCs is blocked (*hm*FCCs are biologically “caged” FCCs) as a consequence of a biosynthetic effort in the (banana) plant, which is seemingly useless in the absence of a further biological role.^85^

Several phyllobilins, such as nonfluorescent type‐I phyllobilins (NCCs), have been shown to be effective antioxidants.^59^ Likewise, YCCs are excellent antioxidants.^98^ Such properties of the amphiphilic phyllobilins are of considerable interest.^59^ DNCCs, the structurally related type‐II analogues, are likely to show basically similar effects. Antioxidants are frequently abundant in plants, especially in ripe fruit,81a and may be crucial for prolonging the viability of cells during senescence^24^ and ripening.^103^

Except for the NCCs and DNCCS, most phyllobilins are classified as photoactive compounds, and may, hence, be considered relevant as cellular metabolites with the capacity to act as light filters. Phyllochromobilins (YCCs, PiCCs, etc.) and their metal complexes could make a particularly important contribution in this respect. Thus, the colors of phyllochromobilins contribute to the pigmentation of leaves^7^ and fruit.^51^ The colors of leaves^104^ and fruit^105^ are considered to be important signals for a variety of animals. FCC fluorescence may, secondly, have a special role as optical brighteners in bananas and banana leaves104a, 106 or act as direct fluorescence signals to animals.^107^

FCCs are specifically efficient sensitizers of singlet oxygen (^1^O_2_): The FCC ***epi*** **‐23‐Me** sensitized light‐induced generation of ^1^O_2_ with a quantum yield of about 0.6.^95^ Clearly, the photochemical capacity of FCCs in sensitizing the formation of ^1^O_2_ may play a physiological role in (senescent or ripening) plant tissue. ^1^O_2_ can function in pathogen defense, as well as acting as a transiently existing signal molecule with messages for receptors in the cell nucleus.^108^ Important signals mediated by ^1^O_2_ are believed to originate from chloroplasts.^109^ Photoexcited FCCs could behave as alternative production sites for ^1^O_2_ in the cytosol. In light of the important roles of the transiently existing ^1^O_2_ 110 in plants,^108^ accumulation of the persistent *hm*FCCs in ripening bananas28a, 85 and in leaves of some other evergreens28b, 74, 76 may indicate a physiologically relevant role of FCCs as ^1^O_2_ sensitizers.^95^ In line with this premise, the formation of the blue fluorescent rings around senescence‐associated dark spots on the peel of ripe bananas, as a result of the local accumulation of *hm*FCCs, may have a function in detaining pathogens and in sustaining the viability of the skin tissue.^85^

Phyllobilins have a broad capacity for acting as ligands for transition‐metal complexes. The pink Chl catabolite (PiCC) **47** binds some transition‐metal ions down to nanomolar concentrations.94a Their metal‐chelating ability94b,94c could be the basis for particular physiological roles of some phyllochromobilins in plants characterized as heavy‐metal accumulators.^111^ Such plants concentrate Zn, Cd, Hg, and Ni ions in their vacuoles, where pigments, tentatively assigned as natural phyllochromobilins, were, indeed, first found.13c The formation of metal complexes may, thus, be likely. Furthermore, tridentate and tripyrrolic transition‐metal complexes were shown to possess druglike properties.^112^ Therefore, the capacity of PiCCs for binding metal ions in a tridentate fashion may be of particular relevance from plant physiological and pharmacological points of view.94b

## 10 Summary and Outlook

**Figure 30:**
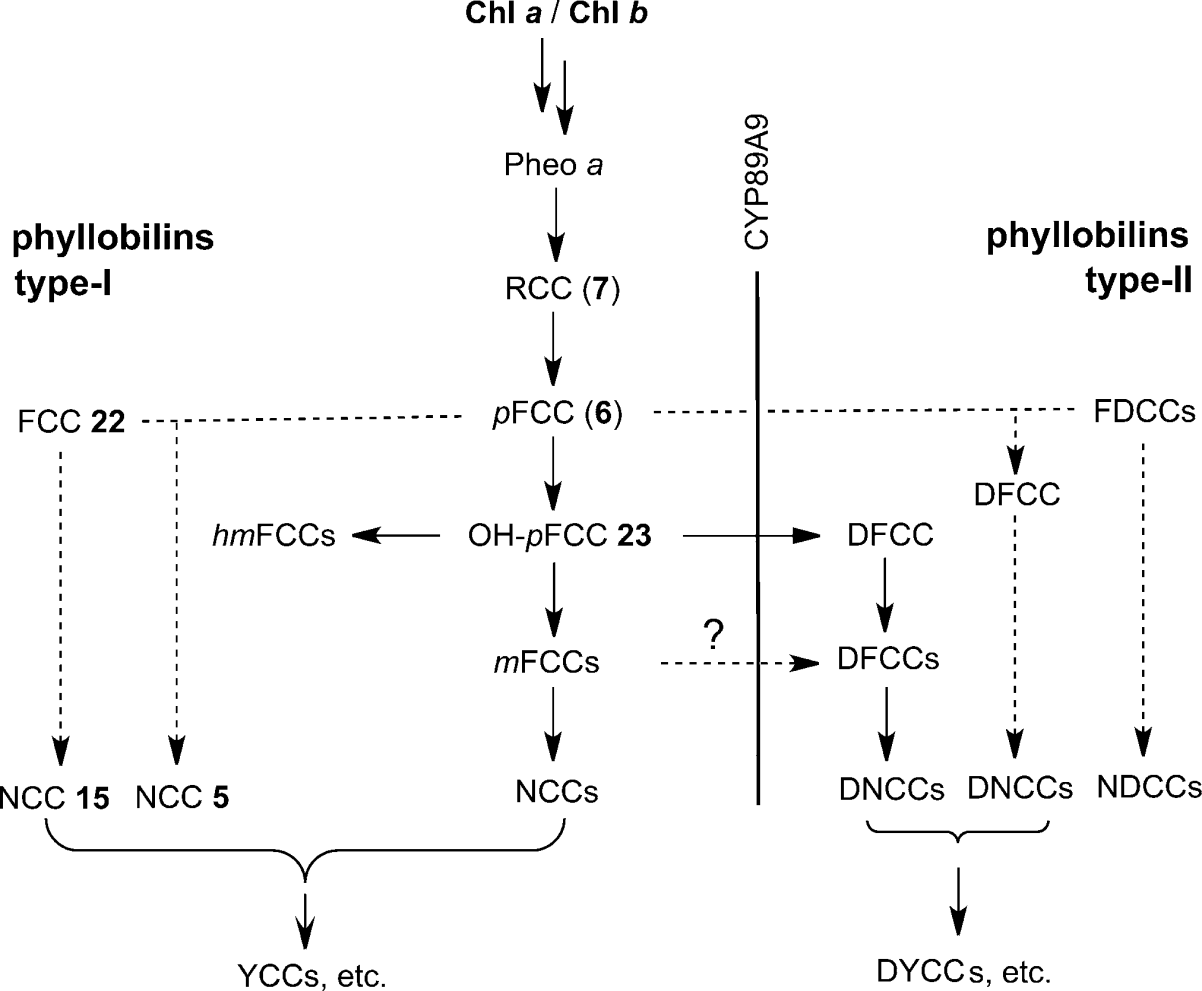
Abbreviated general outline of the PaO/phyllobilin pathway in some higher plants.^10^, ^11^ Chl is broken down in a linear sequence from pheophorbide *a* (Pheo *a*) to primary FCCs (*p*FCCs). Branching out at the stage of FCCs gives several downstream lines of type‐I and type‐II phyllobilins. Colorless phyllobilins (NCCs and DNCCs) accumulate in senescent and ripening plant tissues. The colorless and nonfluorescent NCCs and DNCCs may be oxidized further to yellow (and then pink) phyllochromobilins.

In the past 25 years, research on the identity and on the chemical properties of key Chl catabolites (or phyllobilins) has provided fundamental, structure‐based answers to the question of how Chl is broken down in some higher plants. Indeed, most studies have dealt with the degradation of Chl in angiosperms,9c where natural Chl breakdown is inferred to follow the PaO/phyllobilin pathway. In this pathway, the monooxygenase PaO plays a key role, as it cleaves the chlorin macrocycle and generates the red, native “type‐I phyllobilin”, the barely detectable RCC. Chl breakdown continues rapidly in the chloroplast by furnishing colorless and blue‐fluorescent linear tetrapyrroles (FCCs). In a species‐dependent way either one of the two epimeric primary FCCs (**6**/***epi*** **‐6**) is thereby made. At the FCC stage, the PaO/phyllobilin pathway splits into (at least) two major, and several minor downstream channels (Figure 30). This part of the catabolic pathway is mostly managed by enzymes associated with the cytosol. In some plants, one important second branch produces 1,19‐dioxobilin‐type (or type‐II) phyllobilins by oxidative deformylation of FCCs. Chl breakdown appears to end with the transformation of fluorescent phyllobilins in the acidic vacuole into colorless, and essentially photoinactive, nonfluorescent analogues, which often accumulate in senescent leaves. However, in some plants, the typically short‐lived FCCs are biosynthetically “caged” with complex ester groups and remain fluorescent as persistent hypermodified FCCs (*hm*FCCs). The accumulation of *hm*FCCs in bananas, observable by their bright blue fluorescence, is a puzzling and striking phenomenon that may be useful as a signal for fruit‐eating animals.

Having learned how Chl is broken down to temporarily accumulating colorless catabolites in some higher plants, the question arises logically: What is the fate of the colorless phyllobilins? Are they broken down further in controlled metabolic processes? The recently discovered endogenous formation of phyllochromobilins may play a particular role and hold a possible key to help answer this question. Current work in our laboratories is directed at finding evidence for this.

Chl breakdown has, so far, been analyzed only in a tiny fraction of the vast plant kingdom. In some investigations, naturally senescent leaves of very closely related species, such as the deciduous apple and pear trees^59^ as well as plum and apricot trees^68^ which are all *Rosaceae*, were analyzed and showed a strongly related pattern of colorless phyllobilins. In other plants investigated, such as *A. thaliana* and related *Brassicaceae*, or in the tropical monocots *Musa acuminata* and *Spathiphyllum wallisii*, the PaO/phyllobilin pathway was discovered to produce other types of phyllobilins.

Our investigations have so far been driven by an interest in discovering the most relevant, basic types of phyllobilins in higher plants, rather than exploring the plant kingdom in a biologically systematic way. Clearly, it will be of interest to learn about Chl breakdown in (higher) plants outside the range of the angiosperms. In the specific case of the green alga *Auxenochlorella protothecoides*, the early steps of Chl breakdown are remarkably related to those of the PaO/phyllobilin pathway of higher plants.11a

So far, most studies on bilin‐type Chl catabolites have relied on their analysis in plant extracts. Clearly, besides such eye‐opening in vitro studies, the observation of Chl catabolites in vivo may provide further insight into the effective location of processes relevant for Chl breakdown in the tissue of a leaf or a fruit. Exploratory methodological mass spectrometric^113^ and fluorescence spectroscopic studies^85^ of specific Chl catabolites have begun to pave a more systematic way to in vivo analyses of Chl breakdown.

In a distantly related context, exploratory studies of Chl breakdown in herbivorous protists and insects were recently reported. Thus, in the gut and digestive tract of herbivorous caterpillars, Pheo *a* was detected as the major left‐over product of the ingested Chl.^114^ Furthermore, in the aquatic ecosystem, herbivorous protists were found to degrade (and detoxify) ingested Chl to a nonfluorescent cyclopheophorbide *a* enol.^115^ Clearly, the related question is no less intriguing: What happens to Chl in the metabolism of humans and higher animals.9d, 10

The discovery of hypermodified FCCs is particularly fascinating,28a as is the broad occurrence of type‐II phyllobilins as products of Chl breakdown.^57^, ^58^ These latter 1,19‐dioxobilins and the heme‐derived (hemo‐)bilins share substantial similarity, as they differ (mostly) only by the Chl‐derived “extra” ring E of the type‐II phyllobilins. Important chemical properties of the two lines of bilin‐type natural products can be rather similar. Hence, phyllobilins are candidates for a variety of important biological roles, which are now an apparent domain of the (hemo‐)bilins.^116^ Hence, physiological roles of phyllobilins, for example, as external signals,28a, 117 in intra‐ or intercellular signaling,108b, 118 or in plant defense, can not be discounted.^119^ In this respect, phyllochromobilins may be particularly interesting experimental targets. It is truly remarkable that biological roles of the phyllobilins still wait to be discovered.

Chl breakdown is the remarkable visual sign of leaf senescence and of the ripening of some fruits. Senescence is typically associated with apoptosis, whereas ripening is not. Senescence may be caused by endogenous developmental processes^120^ or by environmental (stress) factors (such as low temperatures or the lack of light,^121^ water, or certain nutrients).^122^ A seasonal combination of several of these factors may be the underlying cause of the apparently synchronized Chl breakdown that is observed in leaves in the fall.101b, 123 Senescence and degreening of leaves may also be induced more locally by external factors (other than the ones listed above), such as mechanical or chemical damages, or by infections with pathogens.^124^ Plants may undergo specific responses, depending upon the means by which senescence is induced.^125^ Ripening should be seen as a basically different developmental process that is typically accompanied by Chl breakdown.11c Hence, it is interesting to test the profiles of the phyllobilins formed in response to the different mechanisms that induce Chl to disappear.

Products of Chl breakdown accumulate in senescent leaves, in developing vegetables, and in ripening fruit. Their value as health‐sustaining nutritional components for leaf‐eating and frugivorous creatures (from insects, snails, birds, to mammals and humans), has hardly been addressed yet.6b, 59, 61, 126 It is tempting, when eating apples, to consider the presence of NCCs in fruit as beneficial to our health as expressed in the old saying: “An apple a day keeps the doctor away”.^59^

Clearly, only the tip of the iceberg of the biological phenomenon of Chl breakdown in higher plants has so far been revealed.6b, 9c, 10, 11 Research on the still‐elusive physiological roles of phyllobilins is mandatory, as are studies of the regulation of Chl breakdown in natural (and artificially induced) senescence and ripening, as well as investigations of the roles and interactions of Chl catabolites during the infection of plants by pathogens. The impact of the colorless phyllobilins (which are abundant in fruit and vegetables) on our health and wellbeing may be revealed by pharmacological studies. Without any doubt, the temporarily abundant remains from natural Chl breakdown leave important traces and have a fundamental impact on the biosphere of our Earth. Similar to the heme‐derived bilins (the hemobilins), phyllobilins will continue to fascinate, and the “Tale of the two Bilins” may take many more years to be told.

## 11 Abbreviations

Chl *a*: chlorophyll *a*
Chl *b*: chlorophyll *b*
Chlide *a*: chlorophyllide *a*
DCC: dioxobilin‐type Chl catabolite
DFCC: dioxobilin‐type FCC
DNCC: dioxobilin‐type NCC
DYCC: dioxobilin‐type YCC
FCC: type‐I fluorescent Chl catabolite
FDCC: fluorescent DCC
NCC: type‐I nonfluorescent Chl catabolite
NDCC: nonfluorescent DCC
Phein *a*: pheophytin *a*
Pheo *a*: pheophorbide *a*
PiCC: type‐I pink Chl catabolite
RCC: red Chl catabolite
YCC: type‐I yellow Chl catabolite

*In memory of Nicholas J. Turro and Philippe Matile*

## Biographical Information

*Bernhard Kräutler studied chemistry at the ETH in Zürich*, *where he received his PhD in 1976 working with Prof. Albert Eschenmoser. After postdoctoral studies with Prof. Allen J. Bard (University of Texas*, *Austin) and Prof. Nicholas J. Turro (Columbia University*, *New York), he returned to the ETH to start his own research group. In 1991 he became Full Professor of Organic Chemistry at the University of Innsbruck, and since October 2015 has been Professor Emeritus. His research interests include chlorophyll breakdown*, the *chemical biology of vitamin B_12_*, as well as *functionalized fullerenes and porphyrinoids*.

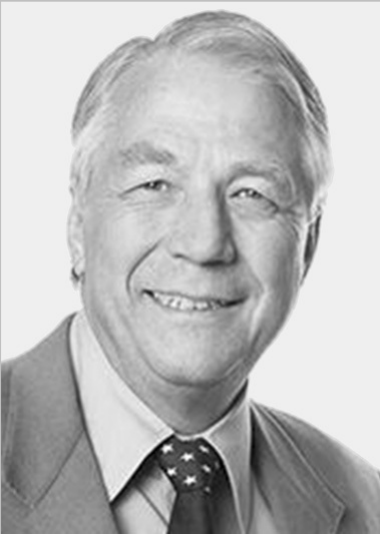
